# Hello, world! VIVA+: A human body model lineup to evaluate sex-differences in crash protection

**DOI:** 10.3389/fbioe.2022.918904

**Published:** 2022-07-19

**Authors:** Jobin John, Corina Klug, Matej Kranjec, Erik Svenning, Johan Iraeus

**Affiliations:** ^1^ Division of Vehicle Safety, Department of Mechanics and Maritime Sciences, Chalmers University of Technology, Gothenburg, Sweden; ^2^ Vehicle Safety Institute, Graz University of Technology, Graz, Austria; ^3^ Chair of Modeling in Engineering Sciences and Medicine, Faculty of Mechanical Engineering, University of Ljubljana, Ljubljana, Slovenia; ^4^ Dynamore Nordic AB, Gothenburg, Sweden

**Keywords:** finite element model, sex-differences, injury assessment, road safety, virtual testing, human body model (HBM), open access, open source (OS)

## Abstract

Finite element Human Body Models are increasingly becoming vital tools for injury assessment and are expected to play an important role in virtual vehicle safety testing. With the aim of realizing models to study sex-differences seen in the injury- and fatality-risks from epidemiology, we developed models that represent an average female and an average male. The models were developed with an objective to allow tissue-based skeletal injury assessment, and thus non-skeletal organs and joints were defined with simplified characterizations to enhance computational efficiency and robustness. The model lineup comprises female and male representations of (seated) vehicle occupants and (standing) vulnerable road users, enabling the safety assessment of broader segments of the road user population. In addition, a new workflow utilized in the model development is presented. In this workflow, one model (the seated female) served as the base model while all the other models were generated as closely-linked derivative models, differing only in terms of node coordinates and mass distribution. This approach opens new possibilities to develop and maintain further models as part of the model lineup, representing different types of road users to reflect the ongoing transitions in mobility patterns (like bicyclists and e-scooter users). In this paper, we evaluate the kinetic and kinematic responses of the occupant and standing models to blunt impacts, mainly on the torso, in different directions (front, lateral, and back). The front and lateral impacts to the thorax showed responses comparable to the experiments, while the back impact varied with the location of impact (T1 and T8). Abdomen bar impact showed a stiffer load-deflection response at higher intrusions beyond 40 mm, because of simplified representation of internal organs. The lateral shoulder impact responses were also slightly stiffer, presumably from the simplified shoulder joint definition. This paper is the first in a series describing the development and validation of the new Human Body Model lineup, VIVA+. With the inclusion of an average-sized female model as a standard model in the lineup, we seek to foster an equitable injury evaluation in future virtual safety assessments.

## 1 Introduction

The Decade of Action for Road Safety 2021–2030 highlights the need to address the global burden of traffic-related injuries, with over 1.35 million people dying and many millions more injured from road traffic crashes annually (World Health Organization, 2018; World Health Organization, 2021). Although vehicle safety systems have improved over the past decades and offer better protection today, not all segments of society are equally protected. Specific demographics, such as women and the elderly, tend to be at higher risk of injury and fatality in vehicle crashes (Bose et al., 2011; Kahane, 2013; Forman et al., 2019; Abrams and Bass, 2020). The detailed analysis of occupant injuries in frontal crashes by Forman et al. (2019) shows that females tend to have a higher risk than males, particularly in the lower extremity, torso, and cervical spine. Another recent study shows that females are at a higher risk for moderate-severity injuries, especially in the extremities, even when differences in crash and vehicle type are considered (Brumbelow and Jermakian, 2022). Sex-differences in injury patterns are also seen in vulnerable road users (VRU) crashes (Leo et al., 2021). The reasons for these sex-differences are not apparent from the field data analyses. However, they serve as an indication that vehicle safety designs are possibly optimized toward the average male, which is the standard representation in vehicle safety assessments today (Linder and Svensson, 2019).

The conventional safety assessment involves evaluating injury responses of the human body in physical tests representing typical crash scenarios. In these physical tests, crash test dummies are used to represent the human body. Crash test dummies are, however, limited in their capability to represent anatomical detail and biofidelic responses, given the requirement for physical robustness and repeatability. In addition, crash test dummies are uniaxial by design, meaning that they are developed for loading in a predefined direction, and biofidelity is restricted to this direction (Perez-Rapela et al., 2021a). By contrast, computational alternatives using Finite Element (FE) analysis provide the means to represent the complex morphology and non-linear material response of the human body. Commonly known as FE Human Body Models (FE-HBM), not only are these models capable of matching human responses (Wu et al., 2017; Chen et al., 2018) but also have the potential to model omnidirectional responses and predict injury at the tissue levels (Iraeus and Lindquist, 2014; Perez-Rapela et al., 2021b). The capabilities of HBMs open avenues to evaluate safety in non-traditional cases (Katagiri et al., 2016; Perez-Rapela et al., 2019) and future seating configurations (Rawska et al., 2020, Rawska et al., 2021; Östh et al., 2020). HBMs are also useful in assessing injury for a wider range of road users, for example, pedestrians and cyclists. With the ongoing modal shift in mobility to sustainable alternatives, bicycles and personal electric vehicles are expected to become more popular (Pucher and Buehler, 2017; Useche et al., 2019). The injury patterns for these road users will be different from those for vehicle occupants (Leo et al., 2019). Given that half of the global deaths in traffic injuries already involve vulnerable road users (World Health Organization, 2018), safety assessments of the future will also need to focus more on this demographic. The advantages of HBMs make them a potential tool of choice for future safety assessments of all types of road users, especially for injury evaluations at the tissue level.

The developments over the past decades in FE software, FE-HBMs, and the advances in computational resources have opened up new opportunities to assess safety virtually. Virtual assessments are expected to complement and increase the robustness of the current safety assessments (Euro NCAP, 2017). However, a few questions need to be considered as FE-HBMs are adopted in safety assessments. First, *what is the optimal level of anatomical detail in HBM required for a virtual assessment?* The currently available models span various levels of detail in their computational definitions. On one end of the spectrum are the detailed FE models of the Global Human Body Model Consortium (GHBMC) and the Total Human Model for Safety (THUMS) v4/v6 that offer detailed representation at the tissue level (Shigeta et al., 2009; Kato et al., 2018; Schap et al., 2019). On the other end are FE models such as THUMS v3/v5 and simplified GHBMC that have coarser mesh and simplified definitions (Schwartz et al., 2015; Kimpara et al., 2016). Higher detail levels, in general, are associated with higher computational times and numerical robustness issues (Lin et al., 2020), while lower details levels may not provide adequate outputs for tissue-based injury assessment. The level of detail, therefore, needs to be informed by the human injuries and countermeasures that will be evaluated in safety assessments. Second, *how much confidence do we have in outputs from an HBM?* In general, this depends on the level of model validation. The repeatability and reproducibility of HBM validations, in particular on the combination of computational hardware and software version used for the assessment, will be required to promote confidence in the outputs from HBMs. Open availability of HBM definitions and their validations will be a step towards addressing this. Finally, *are the current dummy anthropometries an adequate representation of the population to evaluate injuries?* Current dummies represent an average male, a small female, and a large male (Linder and Svensson, 2019). Following the status quo may not be enough to address the sex-differences seen in injury risks. An average female representation has not yet been realized, though it was recommended in the landmark study used to define the anthropometries of the modern dummies (Schneider et al., 1983).

A new lineup of FE-HBMs, called VIVA+, was developed with the motivation of addressing these questions. The models were developed with the intention of finding a balance between the level of detail and the computational effort, paying particular attention to body regions of interest for the analysis of sex-specific differences. The aim of this paper is to report the region-wise kinetics and kinematics of the VIVA+ HBMs in blunt impacts as a first evaluation of the newly developed models. The intention of the validation process presented in this study was to compare the prediction capability of the models with experimental responses and to evaluate the degree to which the model is able to represent the human response under these loading conditions. Furthermore, a new HBM design and development workflow is presented, which can be used to derive models to represent a wider demographic of road users.

## 2 Materials and methods

### 2.1 HBM development workflow

The FE-HBM lineup presented in this paper consists of an average female (50F) and an average male (50M), modeled in a seated vehicle occupant (O) and a standing vulnerable road user (VRU) posture. We implemented an HBM development workflow where all the development was focused on a base model, and the rest of the models in the lineup were derived as tightly linked derivatives, created using mesh morphing. The seated female (50F-O) was selected as the base model.

### 2.2 Anthropometry and geometry for the seated female base model

The geometric definitions used to develop the base model are described in this section. The height and mass of the model was based on the recommendations from the design specifications for adult dummies (Schneider et al., 1983). This study defined the average female stature as 1,620 mm and body mass as 62 kg. Additionally, for the models, the average age of 50 for adults within the European Union (European Commission, 2020) was used as the target age. A template mesh corresponding to an average female, consisting of the outer skin and surfaces of all skeletal parts, similar to (Hwang et al., 2016a; Hwang et al., 2016b) was obtained from the University of Michigan Transport Research Institute (UMTRI). This template mesh is based on several statistical shape models (SSM), among others, (Reed and Ebert, 2013; UMTRI BioHuman: 3D Human Shapes, 2020), for the outer surface, (Shi et al., 2014; Wang et al., 2016), for the ribs and (Klein, 2015; Klein et al., 2015) for the lower extremities. In a second step, the template mesh for the skeletal parts was replaced with a high resolution geometry, from the original VIVA data set. This data set is based on medical images of an average sized 31-year-old female with a stature of 1,616 mm and a body mass of 61 kg (Gayzik et al., 2009, 2011; Östh et al., 2017a). For each bone, the high-resolution geometry was scaled to fit the bone of the template mesh. Finally, the pelvis geometry was updated to an average female according to a pelvis SSM (Brynskog et al., 2021), and the ribcage geometry was updated to a morphed version of the average male generic ribcage presented in (Iraeus et al., 2020). The morphing of the generic ribcage, to an average female ribcage, was based on a ribcage SSM (Shi et al., 2014) and a sternum SSM (Weaver et al., 2014). The high-resolution geometry was used to create the FE mesh described in Section 2.3, while the coarser template mesh was used for morphing in Section 2.4. (Hwang et al., 2016a; Hwang et al.,2016b). In the generation of the UMTRI template mesh the spine curvature was not fully controlled. Thus, the curvature of the thoracic and lumbar spine was re-defined, using the four joint landmarks predicted by the outer shape SSM–OC/C1, C7/T1, T12/L1 and L5/S1—with the additional assumption that the spine follows the curvature of the skin on the back in-between these points. In addition, the cervical spine vertebrae were re-positioned based on a regression model for seated occupants (Reed, 2017).

The interface between the abdominal cavity and subcutaneous adipose tissue was modeled as a simplified abdominal wall, attached to the superior edge of the pelvic innominate bones and the inferior edge of the ribcage. The cutaneous surface of this abdominal wall was estimated by projecting the skin surface inwards using the thickness of the subcutaneous adipose tissue, predicted using a regression model (Holcombe and Wang, 2014). The pleural surface of the abdominal wall was estimated by offsetting the cutaneous surface inwards using the estimated thickness of the abdominal wall. The thickness of rectus abdominis was estimated to be 8 mm and external/internal oblique and transversus abdominis to be 17 mm in total, according to the measurements of middle-aged women (Ota et al., 2012). A simplified pelvic diaphragm was modeled to separate the pelvic cavity from the subcutaneous “flesh.” The thickness was set to 7.2 mm, according to the control group in a study on the dimension of the pelvic floor (Mørkved et al., 2004).

### 2.3 Model definitions

According to the ISO coordinate system, the models were oriented with the *x*-axis pointing forward, the *y*-axis to the left, and the *z*-axis upwards. The H-Point of the model was defined at the center of a sphere approximated to the femur head. The origin of the coordinate system was set at the H-point of the seated models and at the foot sole directly under the H-point for the standing models.

#### 2.3.1 Finite element mesh design

**FIGURE 1:**
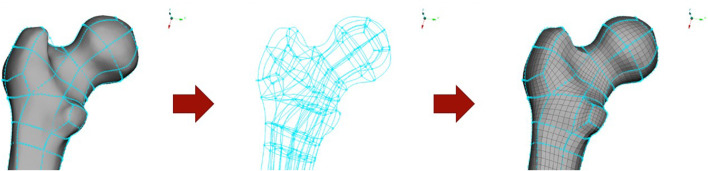
Hexahedral meshing of anatomical components, using hexaboxes approach where the geometry is subdivided into larger hexahedral shapes for control of element quality and flow.

**FIGURE 2:**
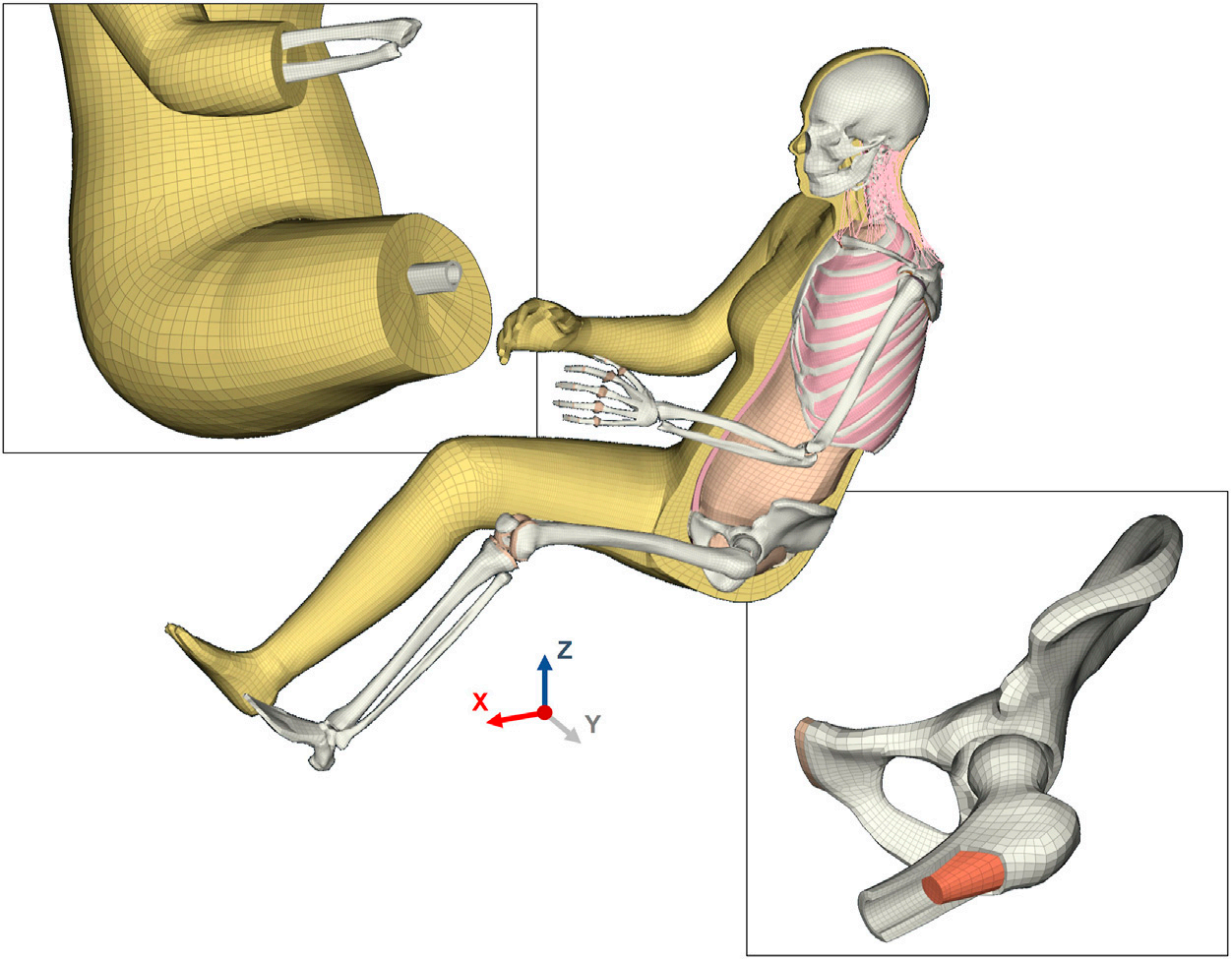
Finite element mesh design of the VIVA + base model. The top-left inset shows the flow of the soft tissue mesh intended to maintain a good mesh quality in all postures ranging from seated to standing. This inset also shows the soft tissue mesh that is nodal connected to the long bones in the lower extremity. The bone-soft tissue interface was not defined with nodal connectivity in the upper extremity but instead defined with contacts. The bottom-right inset is an illustration of the hexahedral mesh in the skeletal structure of the model. The cut-section of the femur shows the solid cortical and trabecular mesh, with cortical bone transitioning from 3-element to 1-element mesh towards the thinner cortical regions in the femur head.

A high-quality hexahedral/quadrilateral element-based mesh was designed on the geometry described above, except for the head, neck, and spine components which were reused from the VIVA model (Östh et al., 2017a). Despite the challenges involved in meshing irregular anatomical shapes with structured hexahedral elements, this was preferred over a tetrahedral mesh considering computational efficiency and control over the structure and size of the mesh after morphing. For first-order (linear) elements, hexahedral elements provide better field approximations than tetrahedral elements and require fewer elements to attain comparable accuracies. An example of the meshing strategy is shown in Figure 1 for the femoral head. The mesh for soft tissues around joints with large rotations (for example, pelvis, elbow, knees) was structured to have a continuous flow so that the elements in the derivative models with different postures would maintain similar mesh quality and sizes (Figure 2). The mesh quality was evaluated using Jacobian, Aspect Ratio, and Warping criteria, and the 100% failure level for solid and shell elements was set at 0.3, 12, and 30, respectively. Finite element preprocessor ANSA versions v20—v22 (BETA CAE Systems, Switzerland) were used for the model development. The mesh of the long bones of the lower extremities (femur, tibia, fibula) was nodally connected to the soft tissue mesh, except in the joint regions (knees and ankles). The soft tissue mesh in the upper extremity, however, is not connected in a similar way because of the relative twist between radius and ulna (Figure 2). Instead, tied contacts were defined for the humerus and surface-to-surface contacts for the radius and ulna to connect bone to soft tissue.

The cortical thickness of the femur shaft was based on a regression model from the same study as the femur SSM (Klein et al., 2015). For cortical thickness in the femoral head, an average of five samples was taken as a reference for cortical thickness (Schubert et al., 2021). For the tibia, the mean cortical cross-sectional areas from Hunter et al. (2019) were taken as target values to define the cortical thickness. In the upper extremity, cortical thickness of the humerus shaft was based on the average thickness reported by Drew et al. (2019) and by Hsu et al. (1993) in the radius and ulna. For all these long bones, the cortical bone was modeled with two or three solid elements over thickness. The hands and feet were defined with simplified mesh using gross approximations of the skeletal geometry

The ribcage was modeled using hexahedral solid elements for the trabecular bone and quadrilateral shell elements for the cortical bone in the same way as for the generic ribcage presented in (Iraeus et al., 2020), but updated to represent an average female. Rib dimensions, cross-section, and cortical bone thickness were initially based on a male dataset (Choi and Kwak, 2011). Next, the dimensions and cross-sections were morphed to represent an average female using a SSM (Shi et al., 2014). As it has been shown that cortical thickness is not significantly different between sexes (Agnew et al., 2018), the cortical thickness was not adjusted. The sternum, based on another SSM (Weaver et al., 2014), was similarly modeled with hexahedral solid and quad shell elements. The intercostal muscles, modeled with hexahedral and pentahedral elements, were given a total thickness according to the same regression model as used for the generic male ribcage previously presented (Iraeus and Pipkorn, 2019). The costal cartilage geometry was based on the original VIVA model (Östh et al., 2017a), with the hyalin part modeled using hexahedral elements and the perichondrium using quadrilateral shell elements.

#### 2.3.2 Material definitions

The material definitions are given in the Supplementary Material A, Table A1. The sources used for the calibration of the material models are indicated in the names of the materials in the model definitions. For stability reasons, fully-integrated elements were used for skeletal parts and under integrated elements for soft tissues. Additionally, to reduce the risk for shear locking in cortical bones modelled using fully-integrated solid element with poor aspect ratio, an enhanced solid element formulation was used. However, as this element formulation is more computational expensive, its use was restricted to body parts where strain will be evaluated for injury risk. Stiffness-based hourglass control was used for all parts except the costal cartilage.

#### 2.3.3 Joints

The modeling of the cervical spine is based on the original VIVA model (Östh et al., 2017b). The knee and hip joint are anatomically modeled with contacting surfaces and ligaments. All other joints are modeled in a simplified way: The intervertebral joints of the thoracic and lumbar spine are modeled as zero-length 1D elements with predefined stiffness based on the VIVA model (Östh et al., 2017a). The shoulder and sternoclavicular joint are also modeled as spherical joints. In the elbow joint, the humerus and ulna are connected using a revolute joint (with the axis through the medial and lateral epicondyle of the humerus). The humerus and radius are connected using a spherical joint (with the center of rotation on the tip of the radius). The radius and ulna are connected with a spherical joint at the distal end (with the center of rotation on the ulnar styloid). The ankle joint is modeled as a revolute joint.

#### 2.3.4 Mass distribution

The male body mass distribution was calibrated based on body region-wise mass and density distribution from a PMHS study (Dempster and Gaughran, 1967). Furthermore, the mass distribution was compared with the anthropometry recommendations for dummies, in which mass was estimated from volume distributions assuming a uniform density throughout the body (Schneider et al., 1983). For the 50th percentile female, however, no comparable data is available. Therefore, information from the development of the EvaRID dummy was used (Carlsson, 2012), where the mass distribution was derived from volume distributions derived from regression models (Young et al., 1983), assuming a uniform density throughout the whole body. Additionally, the inertia properties of the head were based on a review of head properties (Yoganandan et al., 2009).

To meet the target values, the mass density of the flesh was tuned to reach the overall mass. After the first iteration, it was observed that mass was missing in the lower extremities compared to the rest of the body. Thus, the density in this region was set higher than for the rest of the body to rectify this. Also, the seated and standing models did not have exactly the same volume, and therefore two parameters [male/female, standing/seated] were introduced. These parameters are used to scale the densities of the flesh to arrive at the desired mass distributions for all models. The final selected densities and resulting mass distributions compared to the reference values are given in Supplementary Material B, Table B2.

#### 2.3.5 Contact definitions

Contacts are used in the interfaces between bones (joints) and between bones and soft tissues where the meshes are not nodally connected due to function or mesh design. Surface-to-surface contacts are used to model the interaction between soft tissues and hard tissues in the head, neck, upper extremity, torso, and the joints in the lower extremity. Tied contacts are used to tie soft tissues to bone (humerus, sternum, internal organs in the pelvic cavity, and the intervertebral discs and cartilages in the cervical spine). Tied contacts are also defined between abdominal muscles and subcutaneous soft tissue and between the dissimilar mesh of the neck and thorax regions. The details of the contact definitions are given in Supplementary Material B, Table B3. The contacts in the head-neck complex are carried over from the original VIVA model (Östh et al., 2017a). In addition to a surface-to-surface contact definition between the ribcage and the subcutaneous soft tissue, a tied contact was also tested here. The differences in the model responses between the two types of contacts are reported in Supplementary Material D.

### 2.4 Morphing of derivative models

**FIGURE 3:**
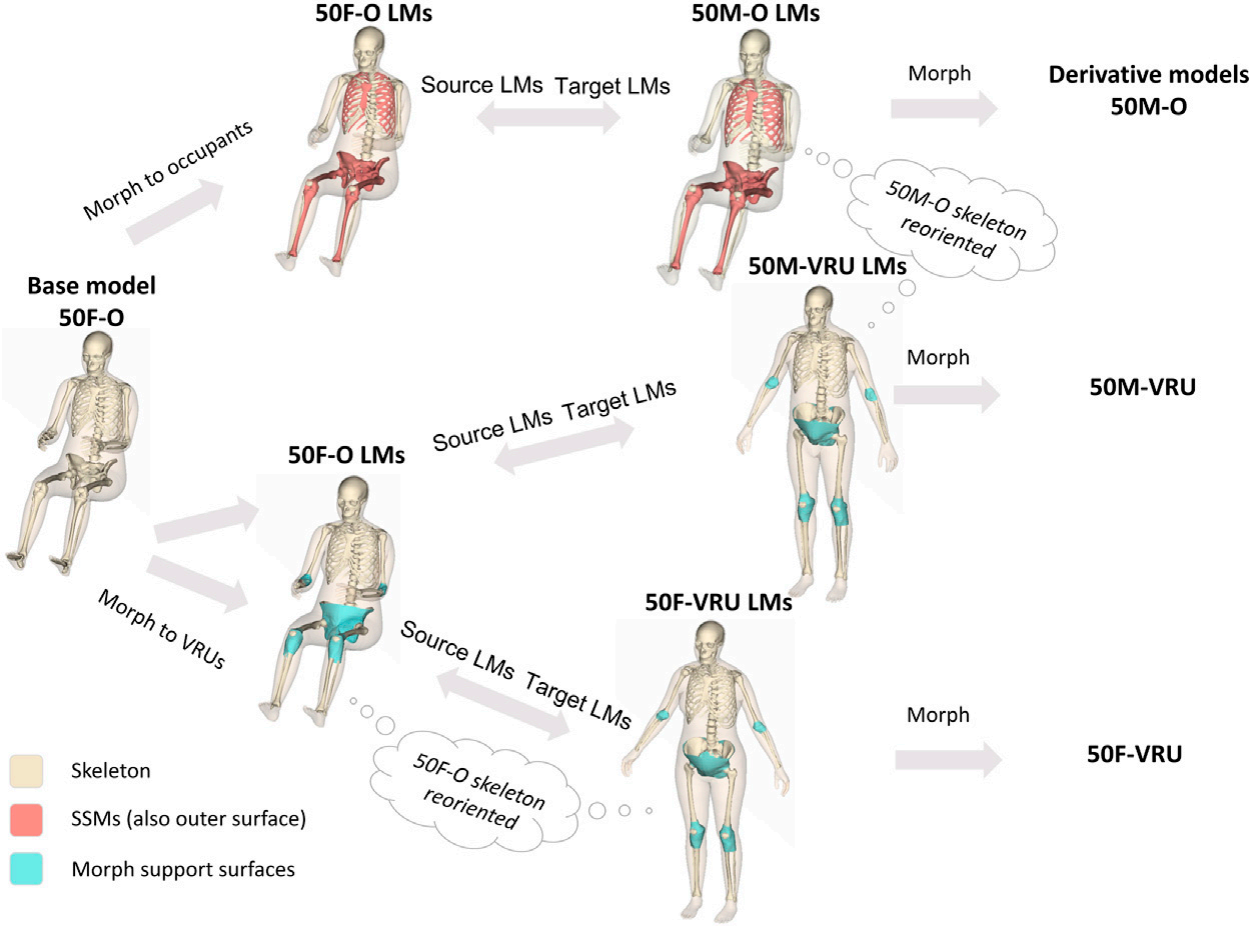
Morphing workflow to create the derivative models. The brown surfaces (including the beige outer surface) represent body parts for which SSMs exist. The beige skeletal surfaces represent body parts that are scaled in other ways or that are repositioned. Blue surfaces represent support surfaces that are pre-morphed and adjusted to guide the morphing in joints that experience large rotations during morphing (LM: Landmarks).

Radial-based function (RBF) interpolation (morphing), based on the same method as in (Hwang et al., 2016b), was used to morph all the base model nodes to the derivative models. In this method, source landmarks were defined using the template mesh of the base model and the template meshes of the derivative models were defined as the target landmarks. The nodal coordinates of all nodes of the derivative models were interpolated based on the coordinates of the source and target landmarks (Figure 3).

As this procedure involves the computationally heavy task of inverting a large matrix, the size of which is based on the number of landmarks (here the number of nodes in the template mesh), it is efficient to split the body into parts and perform the morphing sequentially. Thus, the morphing for all three derivate models followed the same basic steps:

Step 1 Morph the upper and lower extremities separately, following these sub-steps: Morph the skin and bones independently, and save the exterior nodes—Morph the soft tissue between the outer surface and the bones using the morphed exterior nodes from the previous sub -step- Save the morphed nodes in the transition to the torso for later use

Step 2 Second, morph the torso and head, following these sub-steps: Morph the skin and bones separately, and save the exterior nodes—Morph the abdominal wall based on the pelvis and ribcage edges, and save the exterior nodes—Morph the soft tissue based on all saved exterior nodes from Step 1 and Step 2.

Step 3 Finally, assemble all morphed nodes into one file.

The whole morphing code was implemented in MATLAB R2019b (Natick, Massachusetts, United States). The morphing of each derivative model took about 10 min on a laptop (Intel^®^ Core™ i9-9980H). Generally, the morphing code was optimized to produce a good quality mesh for the derivative models. However, patches of elements with poor quality were seen close to joints that underwent large rotations (morphing from seated to standing posture) and around the armpit. The elements in these areas were fixed by manually adjusting nodal coordinates until the element quality criteria were fulfilled for all elements. Additionally, all contacts of the derivative models were fully de-penetrated. These manual fixes took about 4–6 h per derivative model.

The source landmarks for the morphing were a template mesh representing the outer shape and the surface of the skeleton of the 50F-O (the base model). The target landmarks, however, were created slightly differently for the three derivative models, described in more detail below.

#### 2.4.1 Creating target landmarks for the seated male

Similar to the average female, the anthropometry for the average male was based on the design specifications for adult dummies (Schneider et al., 1983). The average male stature was defined to be 1750 mm, the body mass to be 77 kg and the age to be 50 years old. A temple mesh corresponding to this anthropometry was obtained from UMTRI, and similar to the female template mesh, the ribcage (Iraeus et al., 2020) and pelvis (Brynskog et al., 2021) geometries were updated in a second step. The surfaces controlled by SSMs (Shi et al., 2014; Weaver et al., 2014; Klein, 2015; Klein et al., 2015; Brynskog et al., 2021), where sex differences are properly accounted for, are indicated as brown surfaces in Figure 3. All landmarks not controlled by SSMs are indicated as beige surfaces in Figure 3. First of these are the vertebrae of the spine, which were scaled to match the locations of the bony landmarks (the head-neck, C7/T1, T12/L1, and L5/S1 joints) predicted alongside the body surface model. The spine curvature was adjusted using the same method as for the base model. Next, for the bones of the upper extremities and the skull, it was assumed that the size of these parts are proportional to the size of the outer body surface, i.e., they are volumetrically scaled by the dimensions of the outer surface. The exception was the clavicle and the scapula. The clavicle was scaled to match the 50M-O distance between the sternoclavicular and acromioclavicular joints, while the scapula dimensions were scaled to 108% of its original size, based on the ratio of the male to female stature.

#### 2.4.2 Creating target landmarks for the VRU models

The target landmarks of the VRU body surfaces were based on the SSM available at humanshape.org (Reed and Ebert, 2013; UMTRI BioHuman: 3D Human Shapes, 2020). However, as the VRU “individuals” should be similar to the occupant “individuals”, except for the posture, it was decided that the skeletal parts of the VRU models should be reoriented versions of the occupant models. This means that the target landmarks for the VRU skeletal parts are also just reoriented versions of the target landmarks of the occupant models. Similar to the occupant models, the skeletal parts were assembled into the outer shape using the bony landmarks predicted alongside the outer shape (Reed and Ebert, 2013). The same method as used for the occupant models was also used to adjust the spine curvature, but with the additional assumption that the thoracic spine does not change curvature between the occupant and VRU postures. This leads to a rigid transformation of the whole ribcage, including the thoracic spine. The pelvis location and orientation predicted by the outer shape regression model could not be met when comparing to cohorts of middle-aged males or females (Iyer et al., 2016) and another study with a larger mixed sample (Roussouly et al., 2005). As a compromise, the female pelvis was rotated 42° and the male pelvis 39° compared to the occupant models.

The most challenging areas for morphing are the ones undergoing large rotations during the morphing. This includes the areas around the hip, elbow, and knee joints. To get reasonable morphing results, the surfaces enclosing these joints were pre-morphed (using the parts of the skeletal bones) and manually fixed before being added to the target landmarks. These surfaces are indicated as blue surfaces in Figure 3 and are only used when morphing to the VRU derivative models.

### 2.5 Blunt impact validation

Hub impacts at different body regions were selected from literature for the kinetic and kinematic evaluation of the HBMs. The models were evaluated in frontal impacts to the thorax (Kroell et al., 1971; Lebarbé and Petit, 2012) and abdomen (Hardy et al., 2001), lateral impacts to the shoulder (Compigne et al., 2004), thorax, abdomen, and hip (Viano et al., 1989), and back impacts (Viano et al., 2001; Forman et al., 2015). The model response was compared to the available unscaled PMHS data. Force-deflection characteristics were the specific focus to evaluate the overall stiffness of the full HBM. The goal also was to establish load cases to test the overall model performance, when updates are made to the models.

#### 2.5.1 Simulation setup

All simulations were run with LS-DYNA R9.3.1. MPP (ANSYS Livermore Software Technology, California, United States). For consistency, both the 50F and the 50M model responses were analyzed even when experimental data was only available for males. The seated occupant posture versions were used for all load cases except for the lateral Viano impacts, where the standing posture versions were used. The seated models were rigidly rotated around the *y*-axis to match the physical setups, given as transformation in Table 1. The HBMs were not constrained in the hub impacts, and no gravity was applied because the focus of the evaluation was on the first phase (∼20 ms) of the impact, where the whole-body motion is not of relevance.

**TABLE 1:** Hub Impacts used for kinematic and kinetic evaluation (average age, stature, and mass of the subjects are given when available in the literature). The rigid transformation of the HBMs before impact is given in the last two columns. In the velocity column, LS means low speed, MS medium speed and HS high speed.

Load case (Source)		Subjects	Impactor			Impact Location	Transform	
			Dimension [mm]	Mass [kg]	Velocity [m/s]		z_rot	y_rot
Front torso hub Kroell et al. (1971)	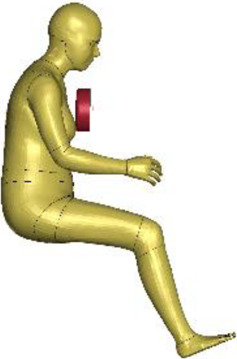	f: 5 (61 years) m: 9 (62 yrs)	152	1.6–23.6	LS: 6.3 HS: 14.3	Midsternal (4th interspace)	0°	23°
Back torso hub Viano et al. (2001)	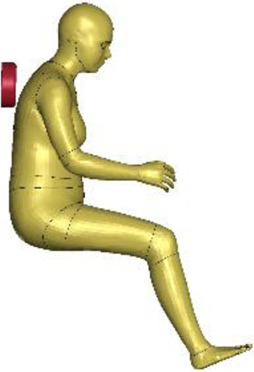	f: 0; m: 8	152	23.4	LS: 4.4 HS: 6.6	Top of impactor aligned with T1 or T6	180°	23°
Back torso hub Forman et al. (2015)	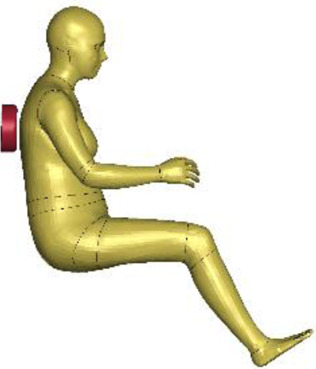	f:0; m: 4 (43 years)	152	97.5	LS: 3.0 HS: 5.5	Center of impactor aligned with T8	180°	12°
Abdominal bar Hardy et al. (2001)	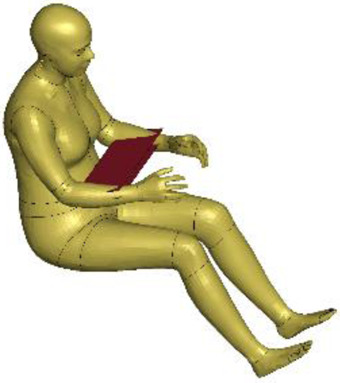	f: 2 (89 years, 165 cm, 54.5 kg)	25	48	LS: 6.3 HS: 9.2	Center of impactor aligned to mid of L3	180°	23°
Shoulder impactor Compigne et al. (2004)	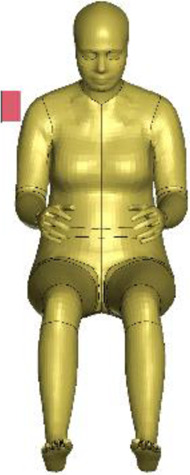	f: 5 (85 years, 57 kg, 164 cm) m: 2 (90 years, 41.5 kg, 164 cm)	Rectangle 150 × 80	23.4	LS:1.5 HS: 6	Glenohumeral joint	90°, 270°	23°
Lateral hub at thorax, abdomen and hip ^∗^ Viano et al. (1989)	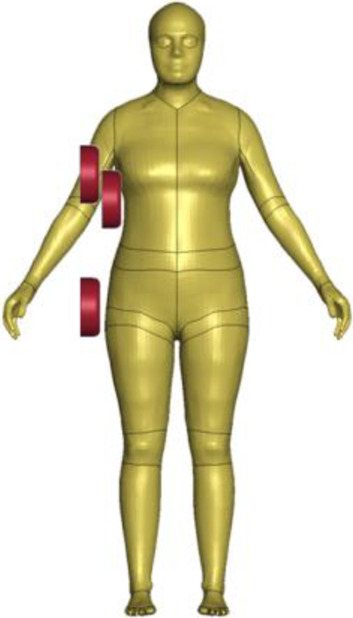	f: 3 (56 years, 57.8 kg, 161.8 kg) m: 11 (53.2 years, 70.25 kg, 173.4 cm)	150	23.4	LS: 4.4 MS: 6.5	Xiphoid process (XP)	330°	0°
					LS: 4.8 MS:6.8	7.5 cm below XP	330°	0°
					LS: 5.2 HS: 9.8	Greater trochanter	270°	0°

^∗^ Impactors are staggered for clarity.

The impactors were modeled with shell elements, using rigid material (MAT_RIGID). The dimensions of the respective impactors are shown in Table 1. All the impactor degrees of freedom were locked except the direction of movement. The impactors were given initial velocity, with magnitude and direction specified in Table 1. The direction was defined such that 0° corresponds to a (frontal) impact in the posterior direction and 90° to the (lateral) impact from the left side (corresponding to the HBM ISO coordinate system). For the back impacts, a stroke limiter was implemented using discrete elements, similar to the physical tests.

A surface-to-surface contact was defined between the relevant body parts of the HBM and impactor. For this contact, a coefficient of friction of 0.3, viscous damping coefficient of 20, the SOFT parameter set to 2, SBOPT to 3, and DEPTH to 5 was applied in all simulations. A minimum timestep of 0.3 μs was defined for all simulations.

#### 2.5.2 Postprocessing

The node histories and contact forces were analyzed with an output interval of 0.1 ms. For the front and back impacts, the chest deflection was defined as the change in displacement between the mid-sternum (fourth rib interspace approximately) and the center of the vertebra at the T8 level. In the lateral hub impacts, the displacement was measured directly on the center of the impactor. For the lateral shoulder impact, the deflection was measured as the displacement between the left and right acromia. In the abdominal impacts, the deformation was measured between the impactor and the center of L3. The simulation outputs were analyzed using Dynasaur Python library in Jupyter notebooks (Klug et al., 2018; Schachner et al., 2022). All outputs were filtered with CFC 180 using the Dynasaur standard function. The output scripts are available in the simulation repository (https://openvt.eu/fem/viva/publications/2022_hello_world). The landmarks used for evaluation are defined as default outputs from the HBM.

### 2.6 Open science

To facilitate replicability and reproducibility of HBM simulations, we adopted certain best practices in Open Science. The model is developed and maintained using the Git version control system. All the model definitions are available under an open license, without encryption. The model documentation is maintained on the same repository as the model and hosted at ReadTheDocs (vivaplus.readthedocs.io). The validations are post-processed with an open-source library (Dynasaur) and cataloged using Jupyter computational notebooks in Python programming language. The simulation setups are made available, enabling verifications and reproducibility of validations across different hardware and software platforms. All the data related to the models and validation are hosted on the OpenVT platform (openvt.eu).

## 3 Results

### 3.1 Human body models

**FIGURE 4:**
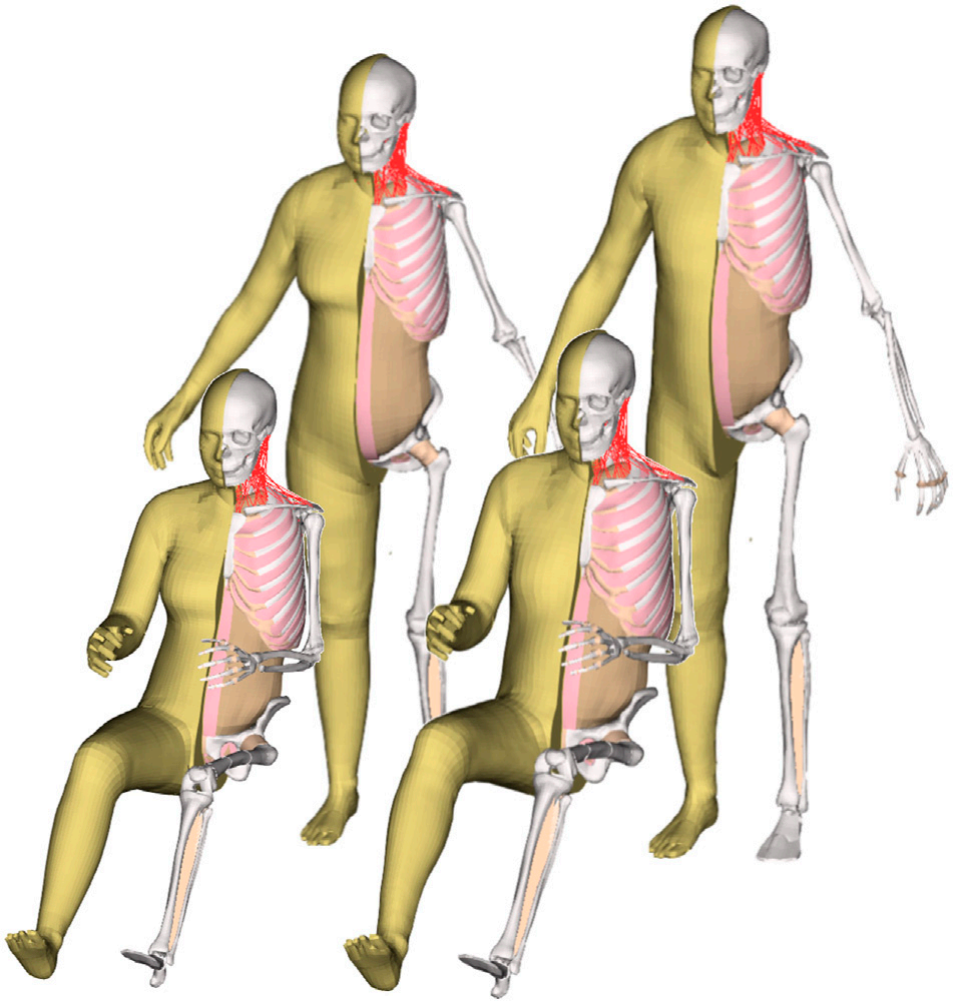
The VIVA + model lineup: the base model of the average female with the derivative male and corresponding standing versions.

The resulting overall model geometries of the derivative models 50M-O, 50M-VRU, and 50F-VRU derived by morphing the base model 50F-O are shown in Figure 4. The comparison of mesh quality distribution of the derivative models with respect to the base model is given in Supplementary Material B, Table B1.

### 3.2 Modular file structure of the developed human body models

**FIGURE 5:**
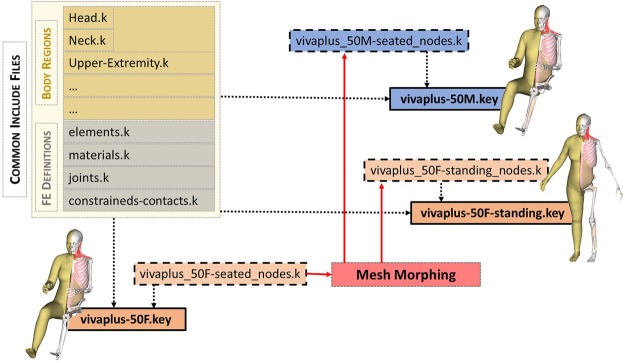
Include structure (LS-DYNA equivalent to functions in programming) of VIVA + models. The model definitions are organized in a modular approach. The main “key” file (shown in boxes with solid borders) refers to the include files. The primary difference between the models is the ‘nodes.k’ include (shown in dashed boxes). The node definitions of derivative models are generated through morphing (standing female and seated male shown for illustration). All the other model definitions–part definitions in the body regions and other finite element definitions–are shared by all the models.

To enable the reuse of model definitions among the base and derivative models, a modular file structure is utilized (an approach similar to functions in programming), as shown in Figure 5. In LS-Dyna, this modular file is known as an “include”. All the models share a set of common include files for FE definitions and part definitions of the body regions. The only difference between the models is the include file defining the node coordinates and the parameter settings to account for posture and sex differences in the main files. This modular structure also facilitates efficient model maintenance for collaborative development. Recommended control cards are also shared with the models. A timestep of 0.3 μs is defined in the recommended control card, giving an initial added mass within the HBM of 0.09 kg (0.12%) for the 50M and 0.12 kg (0.20%) for the 50F. Mass is mainly added to the fibula. If a strain-based assessment is performed for fibula, it is recommended to reduce the timestep.

### 3.3 Kinetic and kinematic evaluation in blunt impacts

The results presented in this section mainly focus on the low severity impacts (with no or low numbers of skeletal fractures), as they were supposed to represent a more realistic loading range for modern vehicles. Simulation results compared to experiments at other loading severities are provided in the Supplementary Material C.

#### 3.3.1 Front impact

**FIGURE 6:**
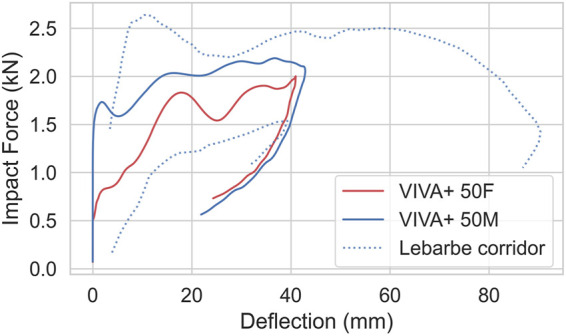
Blunt thorax impact simulations, compared to 4.3 m/s average male corridors from Lebarbé and Petit, 2012.

The blunt thoracic impact simulations are compared to scaled average male experimental corridors in Figure 6 (Lebarbé and Petit, 2012). The female response lies inside the corridor. The initial forces for the male are higher than in the corridor, but inside the corridor after 3 mm deflection. The maximum deflection is on the lower end of the experiments. Forces in the 50F are lower than the 50M, especially at the beginning.

**FIGURE 7:**
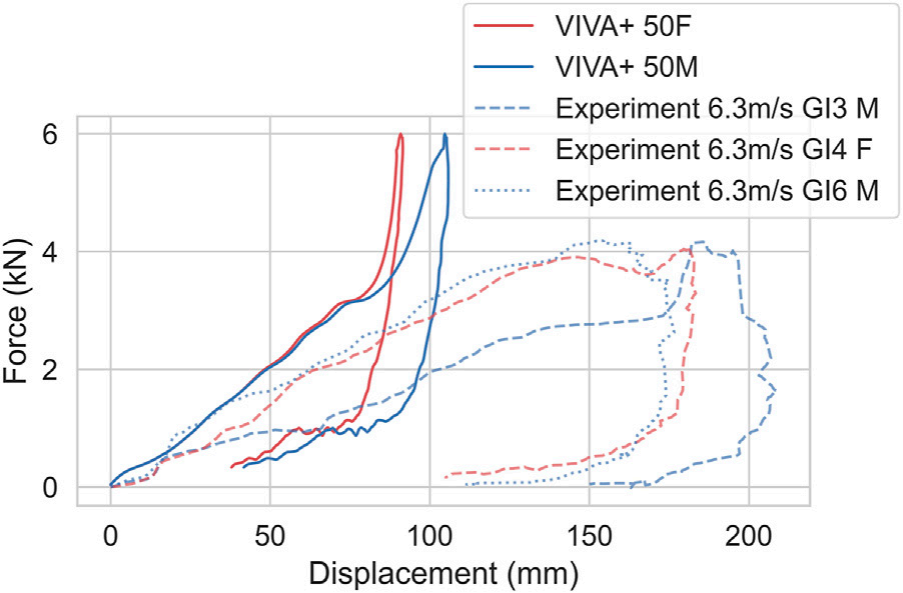
Bar impacts at mid-abdominal region (aligned to L3) at 6.3 m/s, compared to experiments from Hardy et al., 2001. Dashed/dotted lines show experimental responses, red color represents females and blue represents males.

For the abdominal impacts, the simulations follow the upper curve from the experiments up to 40 mm (Figure 7). Maximum deflection is, however, underestimated, and maximum force overestimated. The response of 50F and 50M are very similar, which is in line with the experiments.

#### 3.3.2 Lateral impacts

**FIGURE 8:**
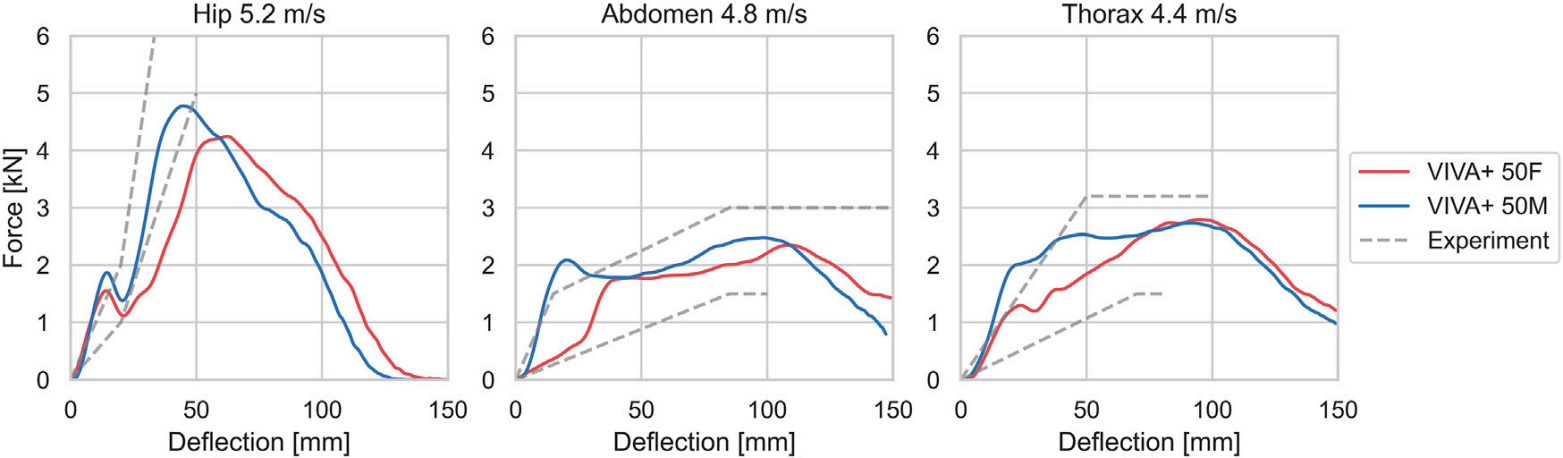
Force-deflection curves from simulations, compared to corridors for low-speed experiments from Viano et al., 1989.

Simulations of blunt lateral impacts at the hip and upper and lower thorax are compared to the non-normalized low impact speed corridors (Viano et al., 1989) are shown in Figure 8. Simulations and experiments are close to each other in respect of the initial slope and the maximum force for the low-speed impacts and are inside the corridor or close to it. The forces for the hip impact of the female are lower than in the experiments. For the abdominal and thoracic impacts, the forces in the simulations with the male are initially higher, but inside the corridor after the initial peak.

**FIGURE 9:**
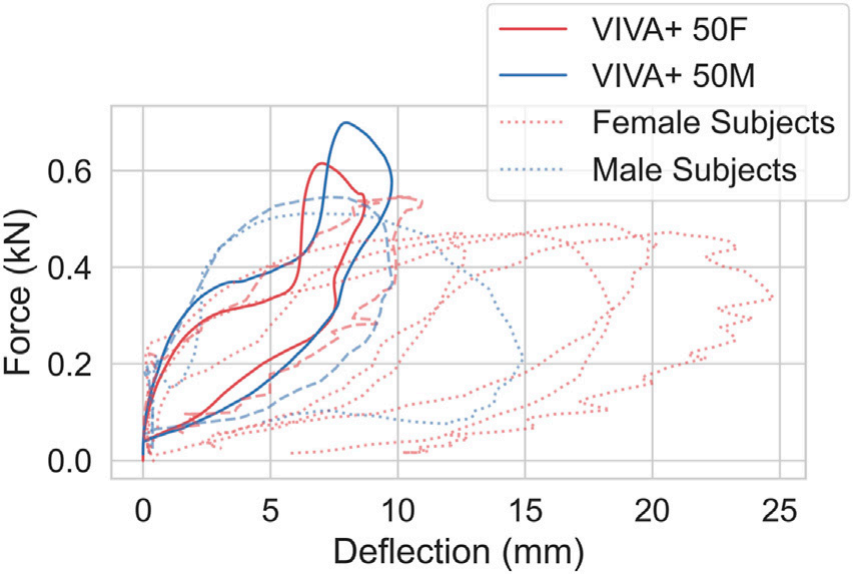
Force versus acromion-to-acromion deflection curves of shoulder impact at 1.5 m/s compared with experimental data from Compigne et al., 2004. Dashed/dotted lines show experimental responses, red color represents females and blue represents males. The experimental PMHS responses close to the model response are shown in dashed line for comparison (red dashed line represents female subject #3 and blue dashed line represents male subject #5).

Force-deflection curves for the simulated 0° shoulder impacts are compared to the experimental results in Figure 9 (Compigne et al., 2004). The 50F and 50M models show similar acromion-to-acromion deflections but different force magnitudes. 50M and 50F responded with approximately 30% and 10% higher forces compared to the PMHS results.

#### 3.3.3 Back impacts

**FIGURE 10:**
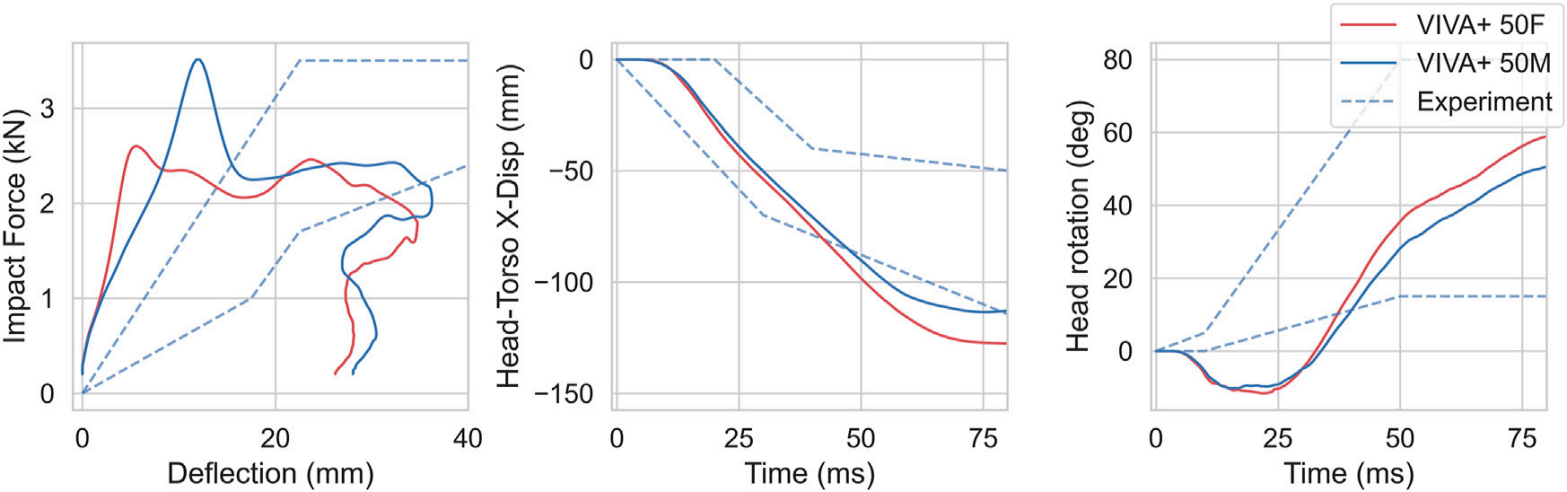
Kinetics and Kinematics for 4.4 m/s blunt impacts at T1, compared with responses from Viano et al., 2001. (left) Impact force-chest deflection response. (center) Head retraction represented by Head x-displacement with respect to T1. (right) Head rotation with respect to T1.

For the back impacts at T1, kinetics and kinematics from the simulations are compared to the experimental responses in Figure 10 (Viano et al., 2001). Higher initial increases in forces are observed in the simulations compared to the experiments. The head rotations with respect to T1 in the simulation show a small initial flexion due to rearward rotation of T1 from the hub impact before the head rotates backward.

**FIGURE 11:**
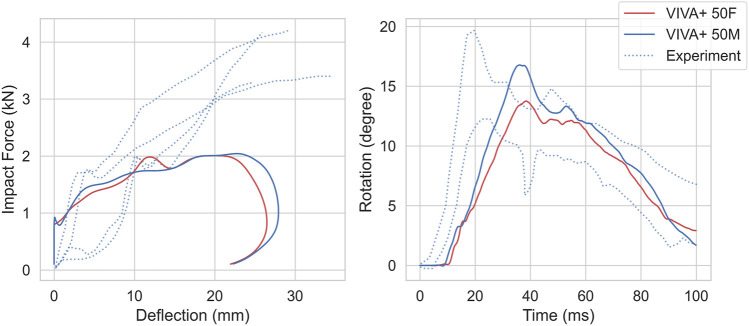
Kinetics and Kinematics for 1.5 m/s impact at T8, compared to Forman et al., 2015. (Left) Force-thorax deformation compared with the four PMHS responses. (Right) T1-L3 Spine Rotation compared with PMHS ± one standard deviation responses. Dotted lines show experimental responses.

For the back impacts at T8 (Forman et al., 2015), the simulation responses are softer compared to the experiments, as shown in Figure 11. The spine rotation in the simulations show the same amplitude and slope as the experiments with a time offset. Differences between 50F and 50M were minor for both kinetics and kinematics. In the experiments, no females were tested.

## 4 Discussion

Computational model development needs to be driven by specific needs and applications, which sets the model’s performance criteria (Cronin, 2011). In the case of the VIVA+ models, we developed the models for tissue-based skeletal injury assessment of road users. With the objective of addressing the needs we perceived for virtual assessment, as described in the Introduction, the VIVA+ models focus on the body regions where we see sex-differences in injuries and attempt to achieve a balance between the amount of model detail and computational overhead. Long bones of the lower extremities, pelvis, and ribs were identified as such body regions. The upper extremities were also assumed to be of interest for future evaluations when considering injuries of lower severity and/or long-term impairments for various types of road users (McMurry et al., 2015; Leo et al., 2019). Therefore, a high-resolution mesh enabling strain-based assessment was implemented in these body regions. It should be noted that the mesh is most likely not converged in terms of stress and strain responses. This resolution is, however, comparable to the state-of-the-art vehicle models, and the mesh size of all these models is governed by today’s computational capacities. All other body regions, including soft tissues, were modeled in a simplified way, enabling realistic overall stiffness and kinematics. As injury assessment of joints was aimed only for the hip, knee, and cervical spine, all the other joints in the model were defined as robotic joints or one-dimensional elements. This combination of detailed and simplified model definition enabled a compromise between computational overhead, numerical stability, and capabilities for skeletal tissue-based injury prediction.

As a step towards achieving higher levels of confidence and credibility in the models, we set up the VIVA+ workflow to enable reproducibility and replicability, both with future versions of the model and other HBMs. Following best practices in Open Science, the model development is done in an automatic version controlled repository, models are available under open licenses, postprocessing is performed with reproducible workflows, and validation setups are made available as open data (Bezjak et al., 2018). Therefore, validations can be repeated by users on different platforms and rerun whenever the model or simulation environment is updated to ensure reproducibility (National Academies of Sciences, Engineering, and Medicine, 2019). To address the question of equitable safety assessment, the VIVA+ models represent both average females and males and thus offer new opportunities to evaluate sex-based differences in injury risks. Furthermore, the unique design and workflow of VIVA+ facilitate derivative representations for other types of road users to reflect the changing patterns of mobility and maintain them as part of the model lineup. We can expect this to expedite the inclusion of a wider proportion of the road user population in safety assessments.

### 4.1 Advantages of the new HBM workflow

As a result of the workflow used in the development, the models benefit from having consistent definitions (for the base 50F-O model and the 50M-O, 50F-VRU, and 50M-VRU derivative models) and enable a direct comparison that is not confounded by differences in mesh resolution or quality. This can be an important aspect when studying sex-differences or posture variations. The tight link between the models also means that all future development and maintenance will be focused mainly on the base model, and the updates are relayed to the derivative models with a minimum effort from the model maintainers. This becomes an important aspect of the open-source sustainability for the models. The derivative models created through this workflow show a slight deterioration of mesh quality as a result of the mesh morphing, but typically at negligible levels, which can be seen in the comparison of mesh quality distributions (Supplementary Material B, Table B1). Deriving standing from seated models and retaining good quality mesh has been considered impractical with the current HBM mesh designs, especially with the large rotation around areas like the pelvis (Hwang et al., 2016a). This was, however, achieved for the VIVA+ models by implementing a unique hexahedral “flow” structuring around the joints with large rotations in the design of the base model. This new approach facilitates the development of additional derivatives representing other types of road users, such as bicyclists and motorcyclists.

### 4.2 Computational cost

The suggested maximum time step of the models is 0.33 µs, limited by the high-resolution mesh of the long bones of the extremities. Using this time step, the added mass (from mass scaling) is approximately 0.1 kg which is reasonable given the total mass of the models (62–77 kg). The model, including about 578,000 deformable elements, has a runtime of 90 min on 32 cores (Intel Xeon Gold 6,130 divided on two CPUs, 56 GB/s Infiniband) for a simulation time of 50 ms. This is computationally more efficient than the current state-of-the-art detailed models but less efficient than the simplified models (For example, the time step of GHBMC M50-OS is 2.8 µs, while that of the detailed M50-O is 28 times smaller at 0.1 µs (Schwartz et al., 2015). However, the computational cost is also dependent on the total number of deformable elements or nodes. For comparison, the GHBMC M50-OS has about 149,000 deformable elements and runs a simulation of 60 ms on 48 cores in 16 min, while the detailed GHBMC M50-O has about 2.2 million deformable elements and runs a simulation of 60 ms on 48 cores in 538 min (Schwartz et al., 2015). If shorter calculation times are required, this can be achieved by rigidifying long bones (similar to the GHBMC M50-OS) for specific load cases, but this then sacrifices the possibility for tissue-based injury assessment for the rigidified parts. If a strain-based assessment of the fibula is performed, the timestep should be further decreased (e.g., to 0.2 µs)

### 4.3 Model responses: Sex-differences

The 50F and 50M models differ only in terms of their geometry and mass distribution. Sex-dependent differences in material properties are not yet implemented in the models. In general, the differences observed between the models seem to be mainly caused by differences in soft tissue dimensions, skeletal size variations, and inertia properties. In the front thorax impact, the female model has an extended initial deformation phase as a result of impacted breast tissue, leading to a lower initial stiffness (Figure 6).

### 4.4 Model responses: Comparison with experiments

The intention of the validation process in this study was to compare the prediction capability of the models with experimental responses and to determine the degree to which the model is able to represent the human response under these loading conditions. All hub impacts were performed using the original postures of the model after applying only rigid transformations. This approach was chosen to enable the users a straightforward replication of the validation simulations with future model updates and other HBMs.

#### 4.4.1 Front impact

For the low speed frontal thoracic hub impacts, the predicted force deflection response was mainly within the PMHS response corridor (Figure 6). Major differences in force-deflection response between the simulations with the 50F and 50M model are observed.

This is most likely caused by the longer distance between impactor and sternum in the females than the males at the time of contact due to shape differences in the soft tissues. Furthermore, the breast tissue softens the initial response. The high-speed results (Supplementary Material C) also matched the PMHS response corridor. In an earlier version a tied contact was used between the ribcage and the subcutaneous soft tissues. This resulted in markedly higher plateau forces ( + 1 kN) and a higher initial spike ( + 1 kN) caused by inertia forces (Supplementary Material D). Based on comparisons to the PMHS response corridors the surface-to-surface contact seems to be a more biofidelic representation of this interface than the tied contact.

For the abdominal impacts, the deviation between experiments and simulations is higher for the low-severity impacts (6.3 m/s) compared to the high severity impacts (9.2 m/s). These deviations are expected to mainly come from the simplified modeling of the organs, where the whole abdominal cavity is modeled with a nearly incompressible Ogden material calibrated to fat tissue, while in reality, parts of the abdominal cavity are compressible. After 40 mm intrusion, the response is dominated by inertia and stiffness effects from the soft tissue, abdominal wall, and abdominal cavity (twice as much kinetic energy as internal energy). However, it should be noted that in the experiments, plenty of injuries were reported (rib fractures and liver injuries in all of them, injuries to the spleen, diaphragm, intestines, tears of the intercostal space in some cases). As the VIVA+ model do not include modeling of neither soft-tissue failure or rib fracture, the model will over predict the stiffness after failure or fracture initiation. For impacts at higher speeds (9.2 m/s), the difference between simulations and tests is small up to 90 mm, which might be caused by the more dominant inertia effects and possibly more biofidelic behavior of the material model used in the higher strain rate range in this test. At higher intrusions, above 40 mm for 6.3 m/s and above 90 mm for 9.2 m/s, the forces are overestimated. The model should therefore not be used to measure forces or intrusions beyond 40 mm or 2 kN. This means that the model should not be used for evaluations after submarining occurs.

#### 4.4.2 Lateral impact

For the lateral blunt impacts at the hip, abdomen, and thorax in low severity impacts, the simulation response was mainly inside the corridors. For hip impacts, a softer behavior of 50F was observed, which is in line with the experimental data; where, however, only one female was tested. The small initial peak during the force increase is most likely an effect of unphysical voids between the soft tissue and the pelvis bones. The abdominal impacts are somewhat mislabeled as these are more in the height of the upper thorax. The observed difference between females and males is caused by the presence of breast tissue where the impactor is contacting the thorax. Also, the higher severity impacts (Supplementary Material C) were mainly inside the corridor. It should also be noted that, as we aim to have a validation catalog that can be easily rerun for new versions of models, the arms of the models were not raised as in the experiments. Inertia effects of the arms could affect the results to some extent.

For the lateral shoulder impacts, the initial slope was well captured. However, the peak force was slightly overpredicted compared to the experiments. The deflection response was close to two subjects in the experiments. This somewhat stiffer behavior is most likely caused by the simplifications in the upper extremities of the current version of the model. The shoulder and the sternoclavicular joints are modeled with robotic joints with a rigid sternum in between. This limits the flexibility and biofidelity of the model in this area, which should be considered in near-side impacts. It might also limit the kinematic biofidelity in VRU impacts, where the body of the VRU is supported by the arms when those are hitting the bonnet. This issue was reported in previous studies for other HBMs, too (Paas et al., 2015). The high speed impacts (Supplementary Material C) showed similar trends, compared to the PMHS results, as the low speed impacts.

Also, for the blunt thorax and shoulder impacts results comparing the earlier version a tied contact between the ribcage and the subcutaneous soft tissues is compared to the current surface to surface contact in Supplementary Material D. Similar trends to the frontal hub impacts can be seen also here, that is, the peak force is overpredicted when using the tied contact, and the current surface-to-surface contact seems to be more biofidelic.

#### 4.4.3 Back impact

In back impacts, the force response was of similar magnitudes when compared to impact at T1 by Viano et al. (2001), but lower after 15 mm when compared to T8 impact by Forman et al. While the deflections are of comparable magnitudes to the Forman experiment, the response is initially stiffer in case of the Viano impact. The deflections are slightly lower for the Viano impact in the T6 low speed impact (Supplementary Material C). This may be explained by the visual measurement method, in which the curvature from ribcage and sternum could potentially obscure the view thereby resulting in higher deformations. In the models, the deflections were measured between the sternum and vertebra. Moreover, replicating the measurement by Viano et al. (2001) is not possible, as it is not clearly defined in the original paper where it is only stated, *“Targets on the impactor, sternum, and spine were used to determine deflection of the chest during impact.”* The model deflections compared well with the more recent experiments by Forman et al., where the boundary conditions were well defined, and deflections were measured using chest bands. However, forces were underpredicted in this loadcase after a deflection of 12 mm. The head-torso rotation kinematics also showed a variation in the first 25 ms when compared to Viano et al. (2001) This difference could be explained by the variations in the initial posture of the subjects. In the simulations, the head placement was more upright compared to the experiments. The higher speed results showed similar trends as for the low-speed impacts (Supplementary Material C).

While the predicted results using the surface-to-surface ribcage to subcutaneous soft tissues contact seemed more biofidelic compared to the tied contact used in an earlier version, the opposite is true for the Forman et al. back impacts. In Supplementary Material D, the results using both these contacts can be compared, and similar to the frontal and lateral hub impacts the peak force increase when using the tied contact. In this case the peak force increases about 1.5 kN, predicting a force level close to the PMHS results. These results might indicate that the subcutaneous soft tissue, in reality, is more connected to the ribcage and spine, than the surface-to-surface contact models, and that different contact modelling strategies may be needed anteriorly and posteriorly.

### 4.5 Model robustness

Several model choices were made to improve model robustness. These were, for example, choosing under-integrated elements over fully integrated elements for soft tissue parts, choosing simple and verified material models over more complex, ensuring that material definitions include sufficient strain hardening to prevent elements from inverting, using nodal connectivity instead of tied contacts were possible, and finally to use a high-quality hexahedral mesh instead of a tetrahedral mesh. The choice of under-integrated elements for soft tissue parts for stability reasons, however, comes with a price, and that is an excess of unphysical hourglass energy. In the load cases analyzed in this study, the ratio of hourglass to internal energy varies between 0.3 and 0.5. However, in other load cases using a car interior, loading a larger portion of the body, this ratio has been somewhat lower. Hourglass models and parameters were iteratively explored during the development stage but did not significantly improve the model results or did lead to stability issues.

### 4.6 Limitations

The VIVA+ models were developed with a specific scope and therefore have several limitations, which should be considered if the model is to be used outside this scope:

1) The upper extremities of the model were modeled in a simplified way, especially the joints. Robotic joints are modeled instead of anatomical joints with soft tissue definitions of ligaments.
2) The thoracic and lumbar vertebrae were modeled as rigid bodies with 1D elements defining the joint properties in between. No strain-based assessment can be performed in this area.
3) Feet and hands were modeled in a simplified way. Injuries in the ankle and wrist cannot be assessed.
4) No element erosion-based failure was implemented in the VIVA+ models. Because of this, peak forces can be overestimated for high severity loading.
5) No internal organs were modeled in detail. Therefore, no injuries can be predicted for the internal organs and the material parameters of the thorax and abdomen were each assumed to be homogenous. This does, however, reduce the complexity of the model (number of elements required for modeling) and increase robustness.

When applying a homogenous density of 1E-6 kg/mm³ for the flesh, the mass for all models would have been too low. This might be caused by voids within the model, especially in the lower extremities (knee capsule, hip capsule, inside long bone shafts, etc.,). Therefore, the density was altered to attain the total targeted mass and mass distributions, leading to slightly different mass densities for flesh in the different body regions—between female and male—as well as between the standing and seated models. The volume of the lower extremity flesh differs between the standing and seated models. This could be the result of deformation of the flesh while seated and have been noted in anthropometric studies (Gordon et al., 2014). In the VIVA+ models, this could also be caused by different samples of volunteers in the regression models describing the shape of the seated and standing models (UMTRI BioHuman: 3D Human Shapes, 2020).

### 4.7 Outlook

This paper presents the first steps taken toward establishing an open science ecosystem for FE-HBMs. The models are hosted on a Git repository at OpenVT (https://openvt.eu/fem/viva/vivaplus). The model users, developers, and researchers can report issues and discuss future development through the Gitlab Issue boards of the repository. The validation reports are maintained on a Validation Catalog, which will be expanded as the models are further developed and validated. The models documentation (https://vivaplus.readthedocs.io/) will be continuously updated, so that data sources and validation load cases are fully documented along with future development of the models. Further derivative models, such as cyclists, pedestrians and e-scooter riders and other anthropometries will be added to the repository in the future. A morphing code will also be made available, which users and developers can use to include model changes into the derivative models. In addition to encouraging collaborative development and research, the steps above will contribute to the open-source sustainability of the models. Furthermore, switches will be included in the model to make it more modular in the future. This will allow the user to switch to more detailed models of the knee ligaments or reduce computational costs by switching the bones of the extremities rigidly and allowing a higher time step size.

The future development of models will be overseen by the VIVA+ steering group [The steering group is a sub-committee within the Open Virtual Testing Organization (OVTO), which hosts and maintains the OpenVT platform]. The steering group will make new releases as updates are made to the models and make recommendations of stable releases for use in design and production. This paper is the first in a series describing the development and validation of the VIVA+ models. Upcoming papers will focus on the validation of occupant and VRU-specific load cases, ranging from validations of isolated structures and body regions to full-scale experiments.

## 5 Conclusion

To summarize, in this paper, we report the development of a new lineup of HBMs aimed primarily for virtual assessment. The models were developed with a balance of anatomical detail in selected body regions of interest and computational efficiency. As a first evaluation of the model, kinetic and kinematic responses were compared to experiments in simple blunt impacts. The front impact to the thorax showed reasonable force-deflection response, while the abdomen forces were overestimated after 40 mm of intrusion. The lateral impact to the shoulder exhibited a slightly stiffer load-deflection response, while the responses in lateral impact to the thorax, abdomen, and hip were close to experimental responses. In the back impacts, the model responded with force magnitudes similar to the experiments at T1 impacts and similar deflection responses at T8 impact. The development of models of this scale and complexity is a continuous process, and this study with simple blunt impacts serves as the first step towards further verification and validation. The development workflow and the Open Science approaches employed for the models make them well suited for collaborative research and makes them accessible to a wider user group. With the inclusion of an average female model as the norm in a model lineup and with the capabilities of the ecosystem to represent a wider range of road users, the VIVA+ models provide the means to enable an inclusive approach in future virtual assessments.

## Data Availability

The models are available at https://openvt.eu/fem/viva/vivaplus. The simulation setups and notebooks/scripts for postprocessing and visualization are available at https://openvt.eu/fem/viva/publications/2022_hello_world.
