# Supplementary material for: Hello, world! VIVA+: A human body model lineup to evaluate sex-differences in crash protection
Source: Front Bioeng Biotechnol. 2022 Jul 19;10:918904. doi: 10.3389/fbioe.2022.918904 (PMC9343945; doi:10.3389/fbioe.2022.918904)
Supplement: Supplementary file 1 [file DataSheet2.pdf]

Supplementary B: Model Definitions

1 Element quality distribution

Table B1: Element quality comparison between base female model and the derivative models

Quality Criteria	Solid	Shell
Jacobian	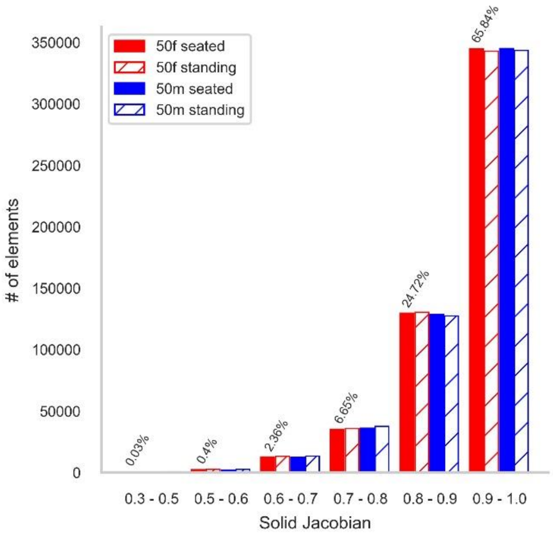	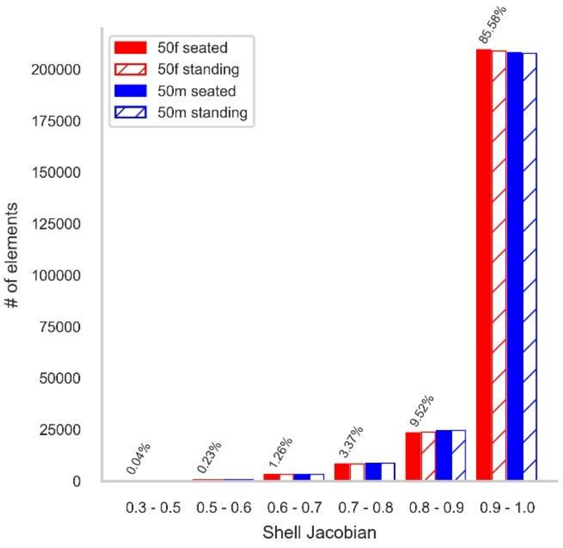

Aspect Ratio	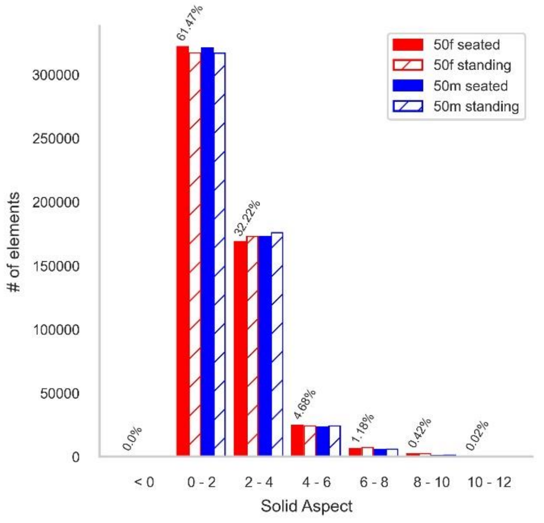	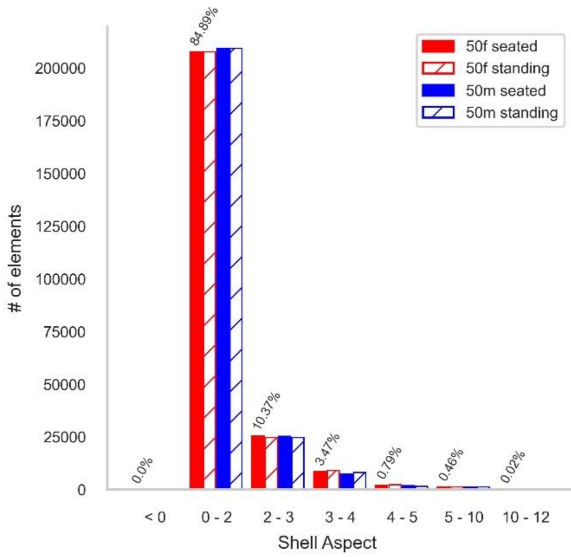
Warping	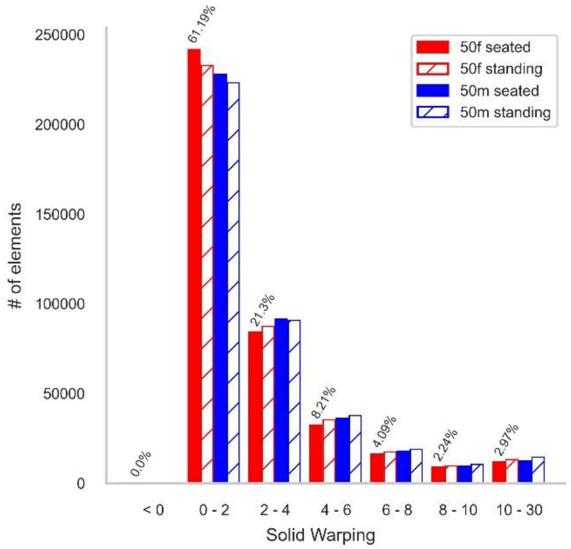	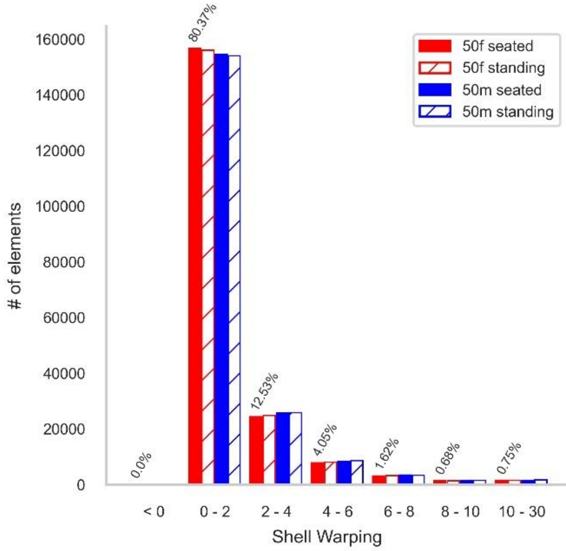

2 Mass distribution of the models

Table B2: Mass distribution of the female and male models, compared with data from literature

	50M				Targets		50F				Targets
	Seated		Standing		Dempster et al.	Schneider et al.	Seated		Standing		References
**Head**	4.4	5.72%	4.4	5.72%			3.9	6.14%	3.9	6.11%	
**Upper Extremity**	8.2	10.71%	8.5	11.03%	9.60%	9.90%	6.5	10.31%	6.0	9.45%	
**Thorax**	16.3	21.27%	15.9	20.65%	22.00%	31.00%	12.8	20.30%	12.9	20.36%	
**Thorax + Abdomen + Pelvis**	38.9	50.64%	38.4	49.99%	52.80%	49.00%	31.9	50.76%	31.8	50.22%	57.89%
**Lower Extremities**	24.2	31.50%	24.2	31.48%	30.80%	34.43%	19.8	31.44%	20.7	32.77%	28.01%
**Thigh**	7.9	10.24%	8.1	10.56%	10.00%		6.5	10.30%	7.0	11.05%	8.32%
**Lower Leg**	3.0	3.94%	2.9	3.74%	4.60%	7.00%	2.5	3.98%	2.4	3.82%	

3. Contact Definition

Table B3: Contact Definitions in the Model

Contact ID	Name	LSDYNA Type	SOFT|SBOPT |DEPTH	FS|FD|VDC
100001	HE_Head_neck_main	AUTOMATIC_GENERAL	1 | - | 2	0 | 0 | 0
200002	NE_Dens_ligaments	AUTOMATIC_SURFACE_TO_SURFACE	1 | - | 2	0 | 0 | 0
200102 - 200607	NE_*_Inf_Disc_TB<br>NE_*_Sup_Disc_TB<br>NE_*_L/R_Inf_Art_Cart_TB<br>NE_*_L/R_Sup_ Art_Cart_TB	TIED_SURFACE_TO_SURFACE	0 | - | 2	-
202100	NE_Discs_ligaments	AUTOMATIC_GENERAL	1 | - | 2	0 | 0 | 0
290001	NE_Tied_neck_to_thorax	TIED_SURFACE_TO_SURFACE	0 | - | 2	-
300000	UX_Single_Surface	AUTOMATIC_SINGLE_SURFACE	1 | - | 2	0.05 | 0.05 | 20
300001	UX_Scapula_Clavicula_Single_Surface	AUTOMATIC_SINGLE_SURFACE	1 | - | 2	0.05 | 0.05 | 20
300010/350010	UX_Humerus_Tied_Left/Right	TIED_NODES_TO_SURFACE_OFFSET	0 | - | 2	-

400001	TX-Torso_Single_Surface	AUTOMATIC_SINGLE_SURFACE	1 | - | 2	0.05 | 0.05 | 20
400002	TX_C_Thorax_Scapula_Clavicula	AUTOMATIC_SURFACE_TO_SURFACE	2 | 3 | 5	0.05 | 0.05 | 20
403505	TX_Contact_tied_sternum_to_soft_tissue	TIED_NODES_TO_SURFACE_OFFSET	-	-
404505	TX_Contact_tied_abdominal_wall_to_soft_tissue	TIED_NODES_TO_SURFACE_OFFSET	-	-
600000	PE_SI-Joint_Tied	TIED_NODES_TO_SURFACE_CONSTRAINED_OFFSET	0 | - | 2	-
600010	PE_Contact_tied_pelvis_to_soft_tissue	TIED_NODES_TO_SURFACE_OFFSET	-	-
700000	LX_Single_Surface	AUTOMATIC_SINGLE_SURFACE	2 | 3 | 5	0.05 | 0.05 | 20
700010	LX_Patella_Surface_to_Surface	AUTOMATIC_SURFACE_TO_SURFACE	2 | 3 | 5	0.05 | 0.05 | 20
903001	ME_C_Whole_body_skin	AUTOMATIC_SINGLE_SURFACE	2 | 3 | 5	0.05 | 0.05 | 20
