# Supplementary material for: Hello, world! VIVA+: A human body model lineup to evaluate sex-differences in crash protection
Source: Front Bioeng Biotechnol. 2022 Jul 19;10:918904. doi: 10.3389/fbioe.2022.918904 (PMC9343945; doi:10.3389/fbioe.2022.918904)
Supplement: Supplementary file 2 [file DataSheet4.pdf]

Supplementary D: Comparison of responses for different definitions of Thoracic contact

Various contacts were tested for the interface between ribcage and the subcutaneous soft tissues during the development of the VIVA+ model. In this supplementary, we report the differences in model responses between two types of approaches in defining this interface: tied contact and surface-to-surface contact. The first one, as the name suggests, ties the soft tissues to the ribcage while the second is the more common definition in HBMs and allow for separation in the interface. between The influence of two contacts on the model responses are compared in this supplementary. The tied contact is contact 400010 in VIVA+ v0.3.0. The tied contact has been removed from v0.3.1 and this interface is included as part of the torso single surface contact (400001). The differences in responses for the thoracic impacts are reported

1.1 Thorax impact

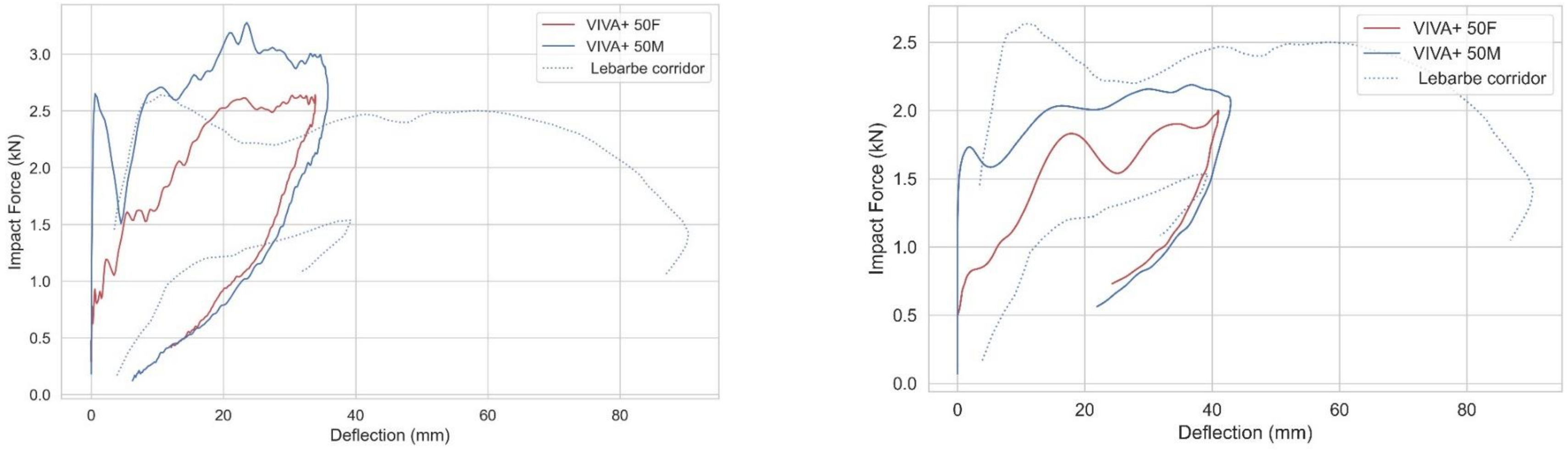

Figure 1: Blunt thorax impact simulations, compared to corridors from Lebarbe et al. (2012). (left) Tied thoracic contact (right) Surface-to-surface contact

2 Lateral Impact

2.1 Torso Hub impact

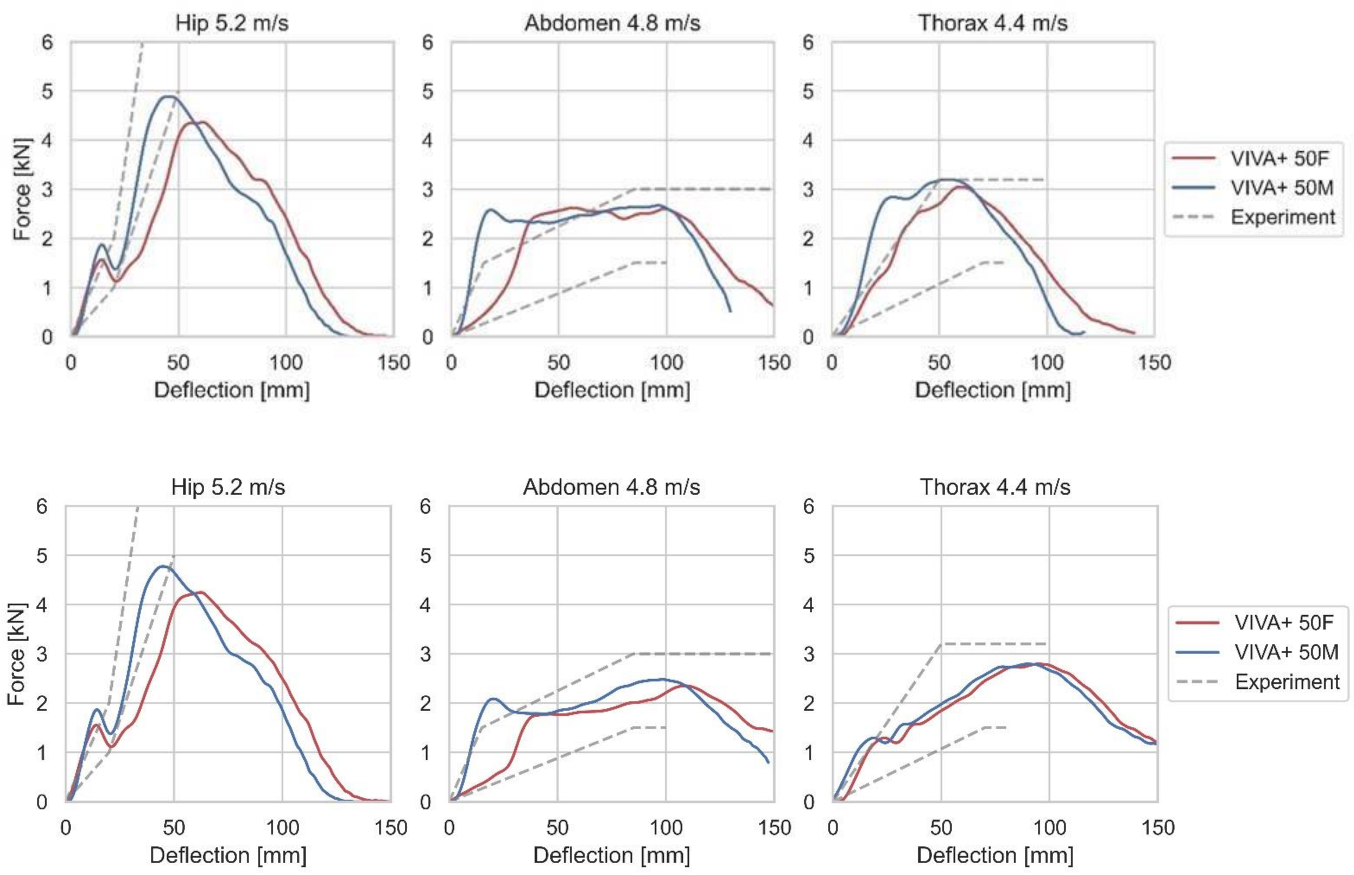

Figure 2: Force-deflection curves for Viano et al. 1989 impacts.). (top) Tied thoracic contact (below) Surface-to-surface contact

2.2 Shoulder impact

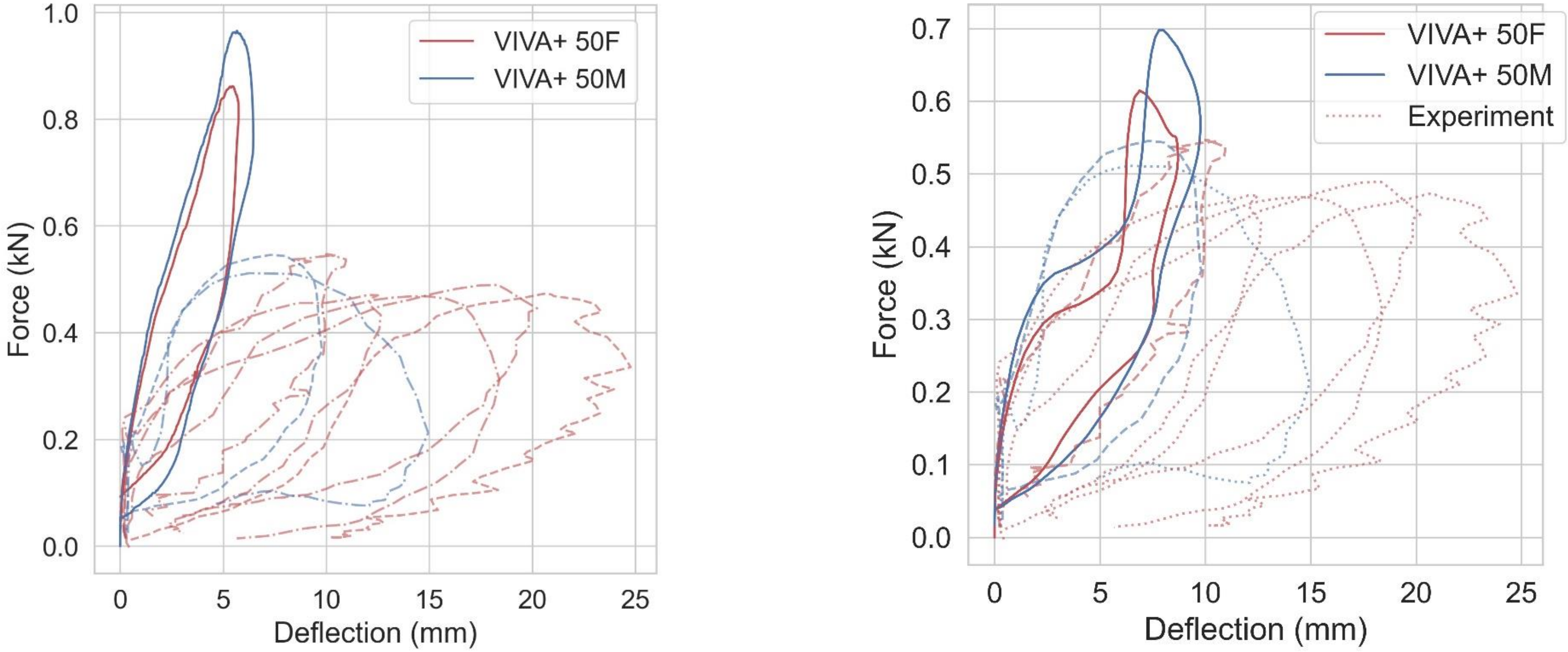

Figure 3: Force-deflection curves of shoulder impact at 1.5 m/s compared with experimental data from Compigne et al. 2004). (left) Tied thoracic contact (right) Surface-to-surface contact

3 Back Impact

3.1 Impact at T1 (Viano et al. 2001)

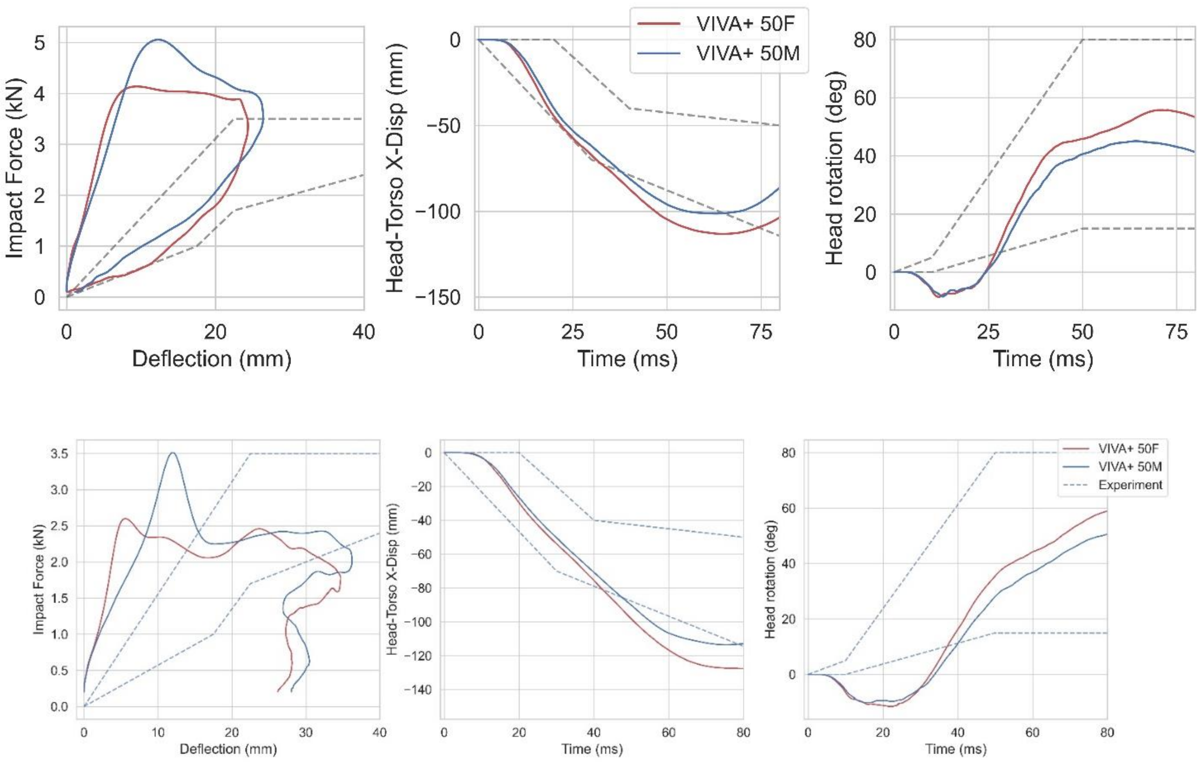

Figure 4: Impact force vs thoracic deflection for Viano et al. 2001 back impact. (top) Tied thoracic contact (below) Surface-to-surface contact

3.2 Impact at T8

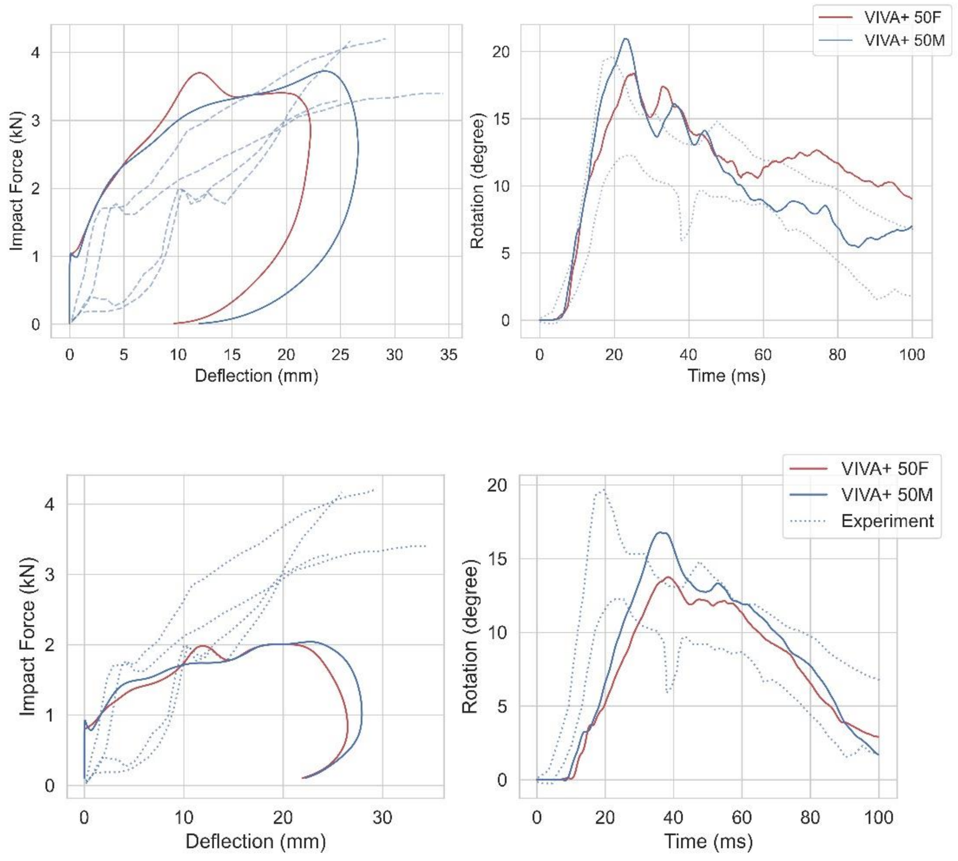

Figure 5: Impact force vs thoracic deflection for Forman et al. 2015 back impact. (top) Tied thoracic contact (below) Surface-to-surface contact
