# Supplementary material for: Hello, world! VIVA+: A human body model lineup to evaluate sex-differences in crash protection
Source: Front Bioeng Biotechnol. 2022 Jul 19;10:918904. doi: 10.3389/fbioe.2022.918904 (PMC9343945; doi:10.3389/fbioe.2022.918904)
Supplement: Supplementary file 3 [file DataSheet3.pdf]

Supplementary C: VIVA+ Responses to blunt impacts at higher severities

1 Frontal impact

1.1 Thorax impact

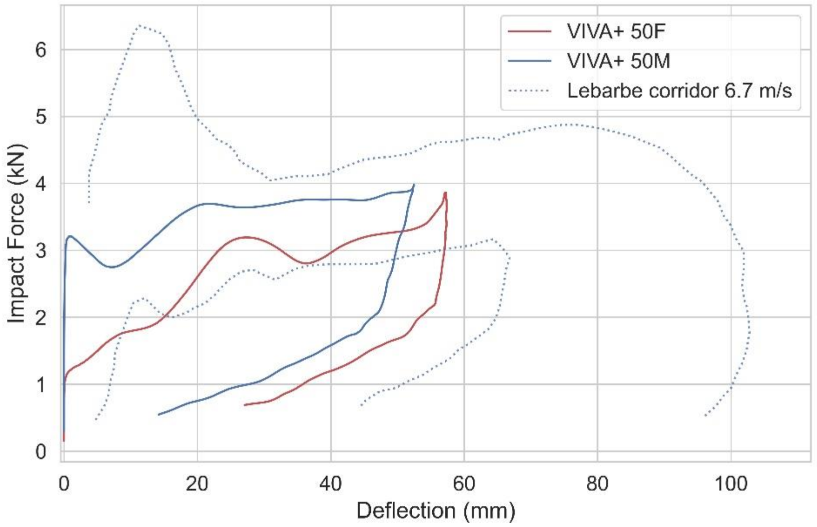

Figure 1: Blunt thorax impact simulations, compared to corridors from Lebarbe et al. (2012) at 6.7 m/s

1.2 Abdomen impact

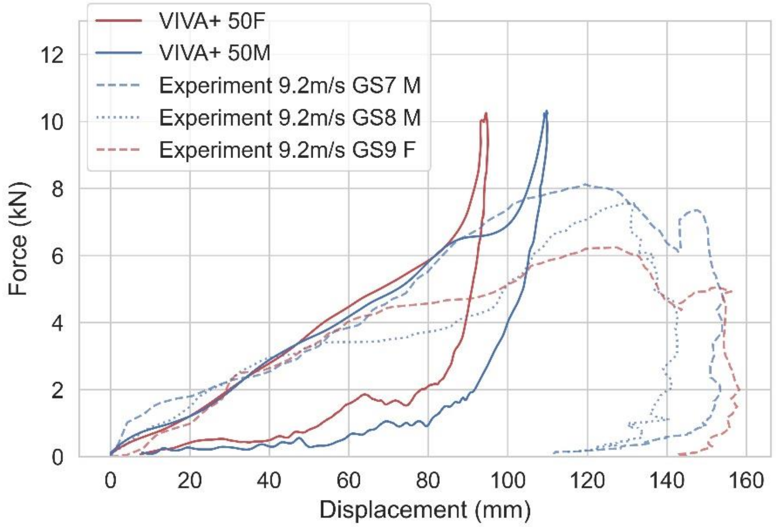

Figure 2: Bar impacts at mid-abdominal region (aligned to mid of L3) at 6.3 m/s, compared to experiments from Hardy et al., 2001

2 Lateral Impact

2.1 Torso Hub impact

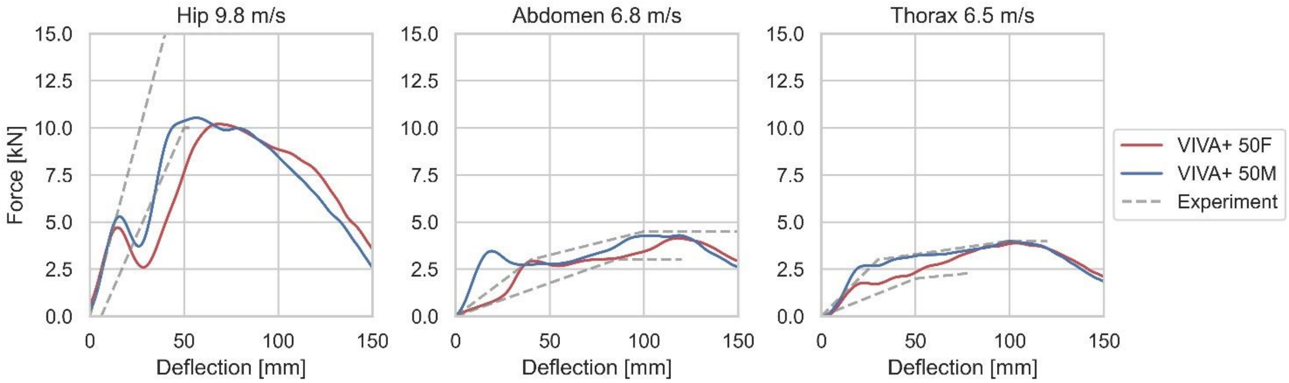

Figure 3: Force-deflection curves from simulations with VIVA+ 50F and 50M model, compared to corridors high-speed experiments from Viano et al., 1989

2.2 Shoulder impact

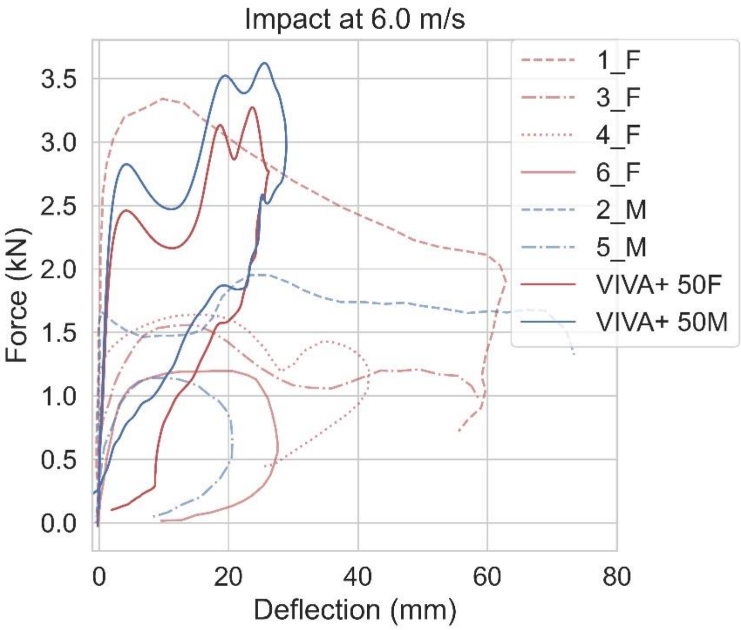

Figure 4: Force-deflection curves of shoulder impact at 6 m/s compared with experimental data from Compigne et al. 2004. The experimental subject number and gender are given in the legend

3 Back Impact

3.1 Impact to the back (Viano et al. 2001)

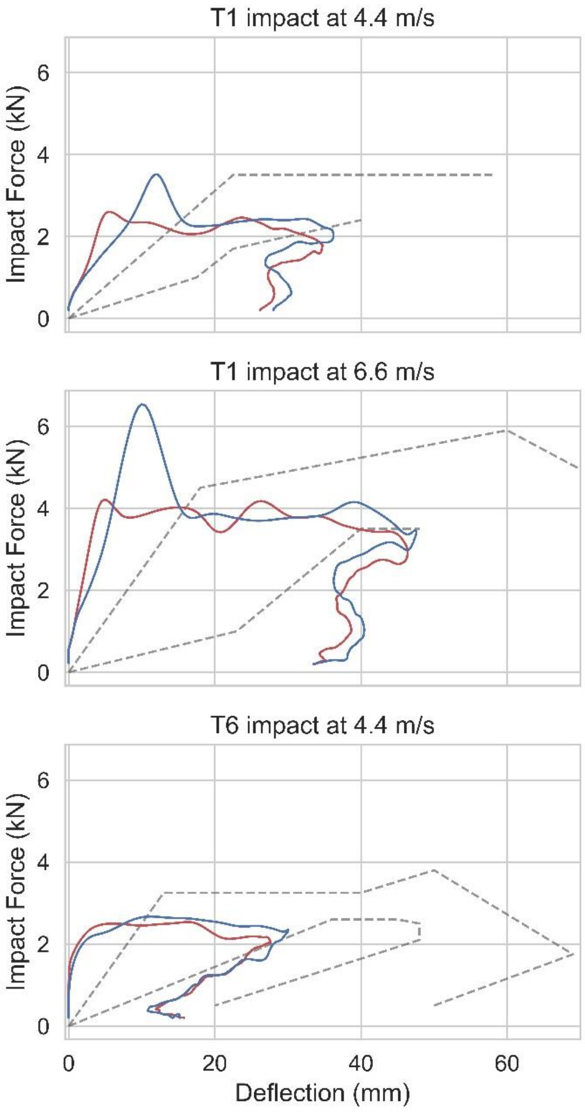

Figure 5: Impact force vs thoracic deflection for Viano et al. 2001 back impact

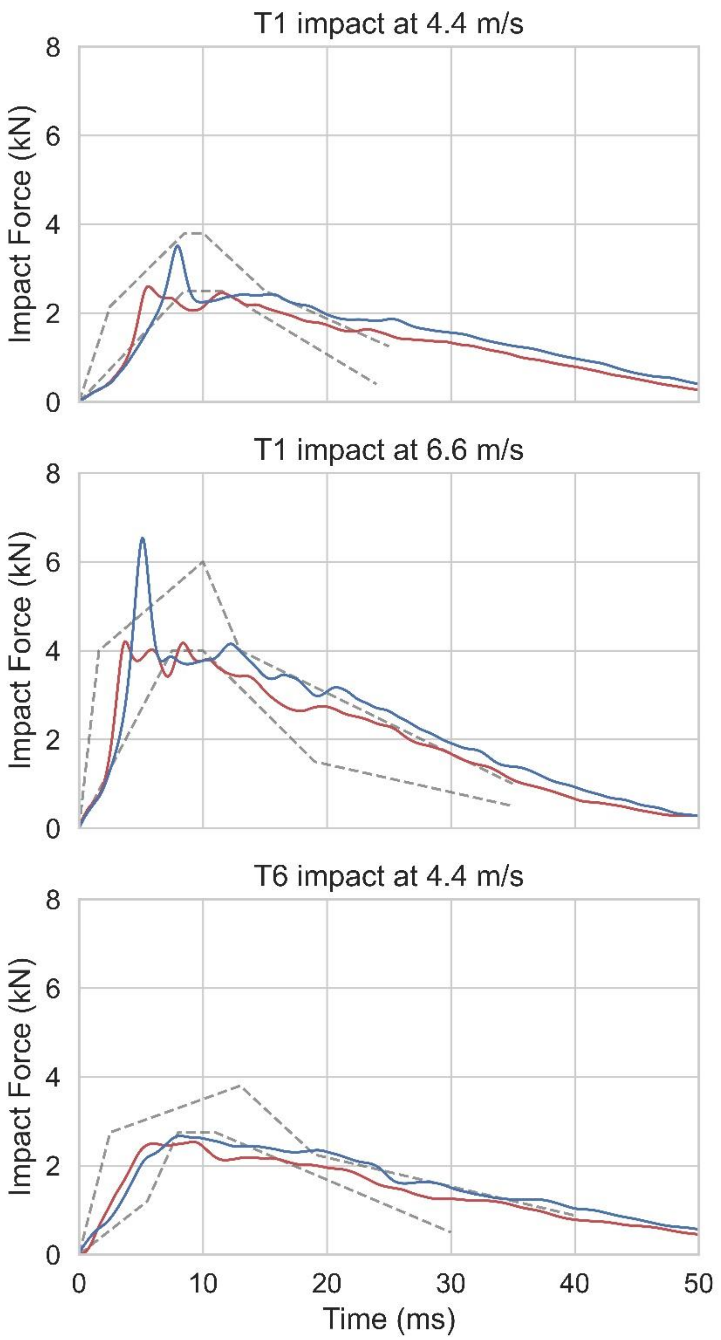

Figure 6. Force-time response for the different impacts reported in Viano et al. 2001

3.2 Impact at T8

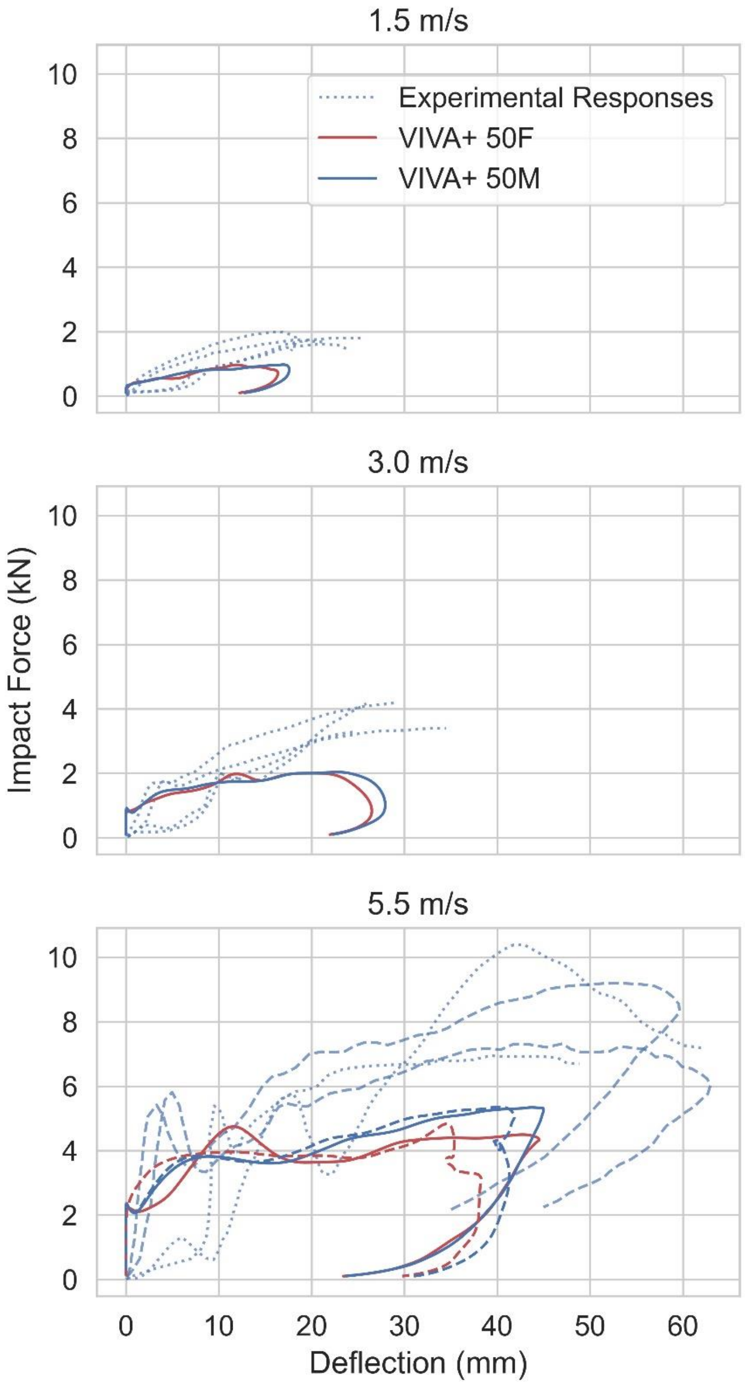

Figure 7: Impact force vs thoracic deflection for Forman et al. 2015 back impact. The dash lines (for experimental and simulation response in the 5.5 m/s subfigure, dashed lines represent the impacts with longer stroke length of 150 mm)

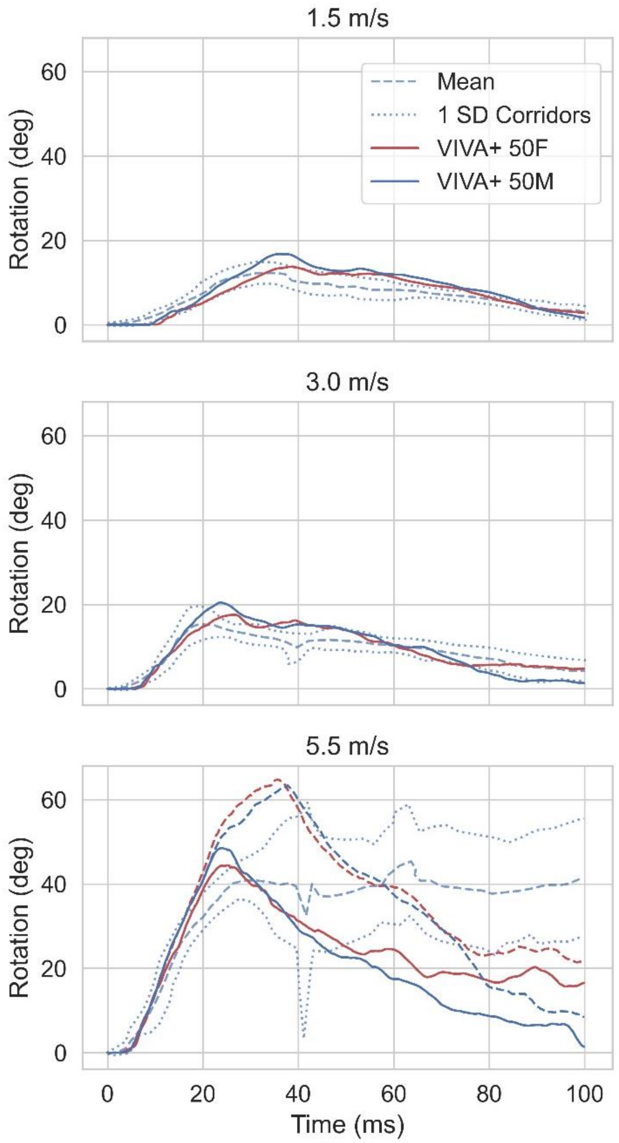

Figure 8: T1-L3 Spine Rotation compared with PMHS ± one standard deviation responses for Forman et al. 2015 back impact (for experimental and simulation response in the 5.5 m/s subfigure, dashed lines represent the impacts with longer stroke length of 150 mm)
