# Supplementary material for: Hello, world! VIVA+: A human body model lineup to evaluate sex-differences in crash protection
Source: Front Bioeng Biotechnol. 2022 Jul 19;10:918904. doi: 10.3389/fbioe.2022.918904 (PMC9343945; doi:10.3389/fbioe.2022.918904)
Supplement: Supplementary file 4 [file DataSheet1.pdf]

# *Supplementary A: Material Definitions*

The definitions in this paper correspond to VIVA+ version 0.3.2. The definitions of the latest version can be found on the online documentation (https://vivaplus.readthedocs.io/)

**Table A1 Supplementary table of the VIVA+ model parts and their parameters sorted by body regions**

| Body Region | Body Part | Material Model | Parameters | Reference | ELTYPE | ELFORM | HG model |
|---|---|---|---|---|---|---|---|
| **Whole Body** | Skin | FABRIC | RO=1E-6<br>BETA=0<br>EA=curve<br>EB=curve<br>PRBA=0.49<br>DAMP=0.05<br>GAB=0.0033 | (Manschot and Brakkee, 1986) | SHELL | 9 | |
| | Flesh | OGDEN_RUBBER | RO=1E-6<br>PR=0.49998<br>ALPHA1=20<br>MU1=3.5E-8<br>BETAI_1=0.006<br>GI_1=8E-7<br>GI_2=1.8E-6<br>BETAI_2=0.05<br>GI_3=2.2E-6<br>BETAI_3=0.6 | (Naseri, 2022) | SOLID | 1 | 900001 |
| **Head** | Skull | PIECEWISE_LINEAR_PLASTICITY | RO=2E-6<br>E=40<br>RO=0.3<br>SIGY=10<br>ETAN=0 | (tuned to Loyd et al., 2014) | SHELL | 2 | |

| | | | | | | | |
|---|---|---|---|---|---|---|---|
| | Oral Cavity | OGDEN_RUBBER | RO=2E-6<br>RO=0.4999<br>G=0<br>ALPHA1=20<br>MU1=3E-8<br>ALPHA2=0<br>MU2=0<br>BETAI_1=0.31<br>GI_1=3E-6 | (Engelbrektsson, 2011) | SOLID | 1 | 900001 |
| **Neck** | Vertebrae - cortical bone | PIECEWISE_LINEAR_PLASTICITY | RO=2E-6<br>BETA=0<br>E=17.1<br>SIGY=1E+20<br>ETAN=0.89668 | (Reilly and Burstein, 1975) | SHELL | 13 | |
| | Vertebrae - trabecular bone | PLASTIC_KINEMATIC | RO=2E-6<br>E=0.495<br>SIGY=1E+20<br>ETAN=0.051 | (Kopperdahl and Keaveny, 1998)<br>(Yoganandan et al., 2006) | SOLID | 4 | - |
| | Neck spine ligaments | FABRIC | RO=1E-6<br>BETA=0<br>EA=1E-6<br>EB=1E-6<br>PRBA=0.3<br>DAM=0.05<br>GAB=individual | (Östh et al., 2016) | SHELL | 9 | |
| | Stylohyoid ligament | CABLE_DISCRERE_BEAM | RO=1E-8<br>E=1.2 | (Zajac, 1989) | BEAM | 6 | |
| | Intervertebral Discs - Nucleus | VISCOELASTIC | RO=1E-6<br>BETA=0.001<br>G0=1.78E-5 | (Yang and Kish, 1988; Iatridis et al., 1996) | SOLID | 1 | 900001 |
| | Anulus ground | HILL_FOAM | RO=1E-6<br>Experimental data | (Panzer and Cronin, 2009) | SOLID | 1 | 900001 |
| | Anulus fibers | FABRIC | RO=1E-6<br>BETA=±45-60<br>EA & EB - individual<br>PRBA=0.3 | (Östh et al., 2016) | SHELL | 9 | |

| | | | | | | | |
|---|---|---|---|---|---|---|---|
| | | | DAMP=0.05<br>GAB - individual | | | | |
| | Vertebra articular cartilage | FU_CHANG_FOAM | RO=1E-6<br>E=0.01<br>DAMP=0.05 | (Östh et al., 2020) | SOLID | 1 | 900001 |
| | Tracheal cartilage | ELASTIC | RO=1E-6<br>E=0.091<br>v=0.4 | (Roberts et al., 1997) | BEAM | 1 | |
| **Neck** | Neck intervertebral muscles | MUSCLE | RO=1E-8<br>Experimental curves | (Östh et al., 2017b) | BEAM | 3 | |
| **Upper Extremity** | Cortical bone | PIECEWISE_LINEAR_PLASTICITY | RO=1.973E-6<br>E=12.516<br>PR=0.2<br>SIGY=0.1<br>ETAN=1 | (Östh et al., 2017a) | SOLID | 2 | - |
| | Trabecular bone | PLASTICITY_COMPRESSION_TENSION | RO=1E-6<br>E=0.829<br>PR=0.3 | (Östh et al., 2017a) | SOLID | 1 | 900001 |
| | Trapezoid & Conoid ligament | SPRING_NONLINEAR_ELASTIC | Experimental curves | (Harris et al., 2000) | DISCRETE | | |
| | Finger interphalanges | OGDEN_RUBBER | RO=1E-6<br>PR=0.49998<br>ALPHA1=16<br>MU1=3E-6 | (Mohammadkhah et al., 2016) | SOLID | 1 | 900001 |
| | Subscapularis muscle | OGDEN_RUBBER | RO=1E-6<br>PR=0.495<br>ALPHA1=13<br>MU1=1.08E-7<br>BETAI_1=0.2<br>GI_1=2.9E-4<br>GI_2=3e-4<br>BETAI_2=100<br>GI_3=1.9E-4<br>BETAI_3=1E+6 | (Mohammadkhah et al., 2016) | SOLID | 1 | 900001 |
| **Thorax** | Ribs and sternum – cortical | PIECEWISE_LINEAR_PLASTICITY | RO=2E-6<br>E=14.7<br>PR=0.3<br>ETAN=0 | (Iraeus et al., 2020) | SHELL | 16 | 900003 |

| | | | | | | | |
|---|---|---|---|---|---|---|---|
| | Ribs and sternum – trabecular | PIECEWISE_LINEAR_PLASTICITY | RO=8.615E-7<br>E=0.04<br>PR=0.45<br>SIGY=0.0018<br>ETAN=0 | (Iraeus et al., 2020) | SOLID | 1 | 900001 |
| | Intervertebral discs | GENERAL_NONLINEAR_6DOF_DISCRETE_BEAM | RO=1E-6<br>Experimental curves | (Östh et al., 2017a) | BEAM | 6 | |
| **Thorax** | Rib cartilage – exterior shells | PIECEWISE_LINEAR_PLASTICITY | RO=1E-6<br>E=0.049<br>PR=0.4<br>SIGY=4.85E-3<br>ETAN=0 | (Iraeus et al., 2020) | SHELL | 2 | 900002 |
| | Rib cartilage - solid | PIECEWISE_LINEAR_PLASTICITY | RO=1E-6<br>E=0.049<br>PR=0.4<br>SIGY=4.85E-3<br>ETAN=0 | (Iraeus et al., 2020) | SOLID | 1 | 403602 |
| | Intercostal external muscle | SIMPLIFIED_RUBBER/FOAM | RO=1E-6<br>Experimental curves | (Östh et al., 2017a) | SOLID | 1 | 900001 |
| | Rhomboideus, Trapezius and Serratus anterior muscle | MUSCLE | RO=1E-8<br>Experimental curves | (Östh et al., 2017a) | BEAM | 3 | |
| | Simplified lungs | LOW_DENSITY_FOAM | RO=5E-7<br>E=0.1<br>BETA=0<br>DAMP=0.5 | (Rater, 2013) | SOLID | 1 | 900001 |
| **Abdomen** | Lumbar Intervertebral discs | GENERAL_NONLINEAR_6DOF_DISCRETE_BEAM | RO=1E-6<br>Experimental curves | (Östh et al., 2017a) | BEAM | 6 | |
| | Simplified abdominal organs | OGDEN_RUBBER | RO=1E-6<br>PR=0.49998<br>ALPHA1=20<br>MU1=3.5E-8<br>BETAI_1=0.006<br>GI_1=8E-7<br>GI_2=1.8E-6<br>BETAI_2=0.05 | (Naseri, 2022) | SOLID | 1 | 900001 |

| | | | | | | | |
|---|---|---|---|---|---|---|---|
| | | | GI_3=2.2E-6<br>BETAI_3=0.6 | | | | |
| | Abdominal muscle | OGDEN_RUBBER | RO=1E-6<br>PR=0.49998<br>ALPHA1=16<br>MU1=3E-6 | (Mohammadkhah et al., 2016) | SOLID | 1 | 900001 |
| **Pelvis** | Pelvis - cortical bone | PLASTICITY_COMPRESSION_TENSION | RO=2E-6<br>E=10.84148<br>PR=0.3 | (Kemper et al., 2008) | SHELL | 16 | 900003 |
| | Pelvis - trabecular bone | PIECEWISE_LINEAR_PLASTICITY | RO=3.45E-7<br>E=0.0236<br>PR=0.2<br>SIGY=1<br>ETAN=0 | (Dalstra et al., 1993) | SOLID | 1 | 900001 |
| | Pubic symphysis | HYPERELASTIC_RUBBER | RO=1.2E-6<br>G=0<br>PR=0.495 | (Li et al., 2006) | SOLID | 1 | 900001 |
| | Sacroiliac Joint | OGDEN_RUBBER | RO=1.2E-6<br>PR=0.495<br>ALPHA1=2<br>MU1=3.224 | (Miller et al., 1987) | SOLID | 1 | 900001 |
| | Hip joint ligament – shell | ELASTIC | RO=1E-6<br>E=0.1368<br>PR=0.45 | (Hewitt et al., 2001) | SHELL | 16 | |
| | Hip joint ligament – beam | GENERAL_NONLINEAR_1DOF_DISCRETE_BEAM | RO=1E-6<br>Experimental curves | (Ito et al., 2009) | BEAM | 1 | |
| | Pelvic cavity tissue | OGDEN_RUBBER | RO=1E-6<br>G=0<br>PR=0.4999983<br>Alpha=20<br>MU1=3E-8 | (Engelbrektsson, 2011) | SOLID | 1 | 900001 |
| | Pelvis floor muscle | ELASTIC | RO=1E-6<br>E=0.15<br>PR=0.4 | (Meyer et al., 1998) | SHELL | 16 | |
| **Lower Extremity** | Femur - cortical | PLASTICITY_COMPRESSION_TENSION | RO=1.8E-6<br>E=7.21 - 16.38<br>PR=0.3 | (Schubert et al., 2021) | SOLID | -2 | |

| | | | | | | | |
|---|---|---|---|---|---|---|---|
| | Femur – trabecular | FU_CHANG_FOAM | RO=2.7E-7<br>E=0.974<br>DAMP=0.05 | (Enns-Bray et al., 2018) | SOLID | 1 | 900001 |
| | Tibia and Fibula – cortical | PLASTICITY_COMPRESSION_TENSION | RO=2E-6<br>E=22.2<br>PR=0.3 | (Reilly and Burstein, 1975) | SOLID | 2 | |
| **Lower Extremity** | Tibia – trabecular | PLASTICITY_COMPRESSION_TENSION | RO=1E-6<br>E=0.829<br>PR=0.3 | (Ding, 2000) | SOLID | 1 | 900001 |
| | Knee cartilage | OGDEN_RUBBER | RO=1E-6<br>PR=0.49<br>ALPHA1=2<br>MU1=0.012 | (Robinson et al., 2016) | SOLID | 1 | 900001 |
| | Meniscus | OGDEN_RUBBER | RO=1E-6<br>PR=0.49<br>ALPHA1=2<br>MU1=0.114 | (Peña et al., 2006) | SOLID | 1 | 900001 |
| | Knee ligaments | SPRING_NONLINEAR_ELASTIC | Experimental curves | (Kunitomi et al., 2017) | DISCR. | | |
| | Patellar ligament | SPRING_NONLINEAR_ELASTIC | Experimental curves | (Müller et al., 2004) | DISCR. | | |
| | Quadriceps femoris muscle | SPRING_MUSCLE | Predefined curves | (Mukherjee et al., 2007) | DISCR. | | |
| | Crural interosseous membrane fibers and proximal tibiofibular ligaments | ELASTIC | RO=1E-6<br>E=1.17<br>PR=0.3 | (Minns and Hunter, 1976) | BEAM | 2 | |
| **Rigid parts** | Skull, mandible, teeth, occipital condyles | RIGID | | | | | |
| | Hyoid bone | RIGID | | | | | |
| | Clavicle, Scapula, Wrist bones | RIGID | | | | | |
| | Thoracic and Lumbar vertebrae | RIGID | | | | | |
| | Patella, Calcaneus, Talus, Tarsal bones | RIGID | | | | | |

* The naming of material models is taken from LS-DYNA keyword library.

** ELTYPE, ELFORM and HG models are not acquired from references.

**Table A2 Definitions of parameters used in Table A1**

| Parameter | Definition | Units |
|---|---|---|
| RO | Mass density | $kg/mm^3$ |
| E | Young's modulus. | GPa |
| PR | Poisson's ratio. | / |
| G | Shear modulus | GPa |
| G0 | Short-time shear modulus | GPa |
| BETA | Material angle for AOPT=3 | degrees |
| ALPHA1 | alpha-1, first exponent. | / |
| ALPHA2 | alpha-2, second exponent. | / |
| MU1 | mu-1, first shear modulus. | GPa |
| MU2 | mu-2, second shear modulus. | GPa |
| GI_1 | Shear relaxation modulus for the 1st term | GPa |
| GI_2 | Shear relaxation modulus for the 2nd term | GPa |
| GI_3 | Shear relaxation modulus for the 3rd term | GPa |
| BETAI_1 | Decay constant for the 1st term | / |
| BETAI_2 | Decay constant for the 2nd term | / |
| BETAI_3 | Decay constant for the 3rd term | / |
| SIGY | Yield stress | GPa |
| ETAN | Tangent modulus | GPa |
| EA | Young's modulus in longitudinal direction | GPa |
| EB | Young's modulus in transverse direction | GPa |
| PRBA | Minor Poisson's ratio in BA direction. | / |
| PRCA | Major Poisson's ratio in CA direction | / |
| DAMP | Rayleigh damping coefficient | / |
| GAB | Shear modulus in AB direction | GPa |

**Table A3 Hourglass models definitions**

| HG model | Description | HG control type (IHQ) | Parameters |
|---|---|---|---|
| 403602 | Hourglass model for rib cartilage | 2 | QM=0.1, Q1=1.5, Q2=0.6, QB/VDC=0.1, QW=0.1 |
| 900001 | Default hourglass model for solids with exact volume integration | 5 | QM=0.1, Q1=1.5, Q2=0.6, QB/VDC=0.1, QW=0.1 |
| 900002 | Default hourglass model for shells with exact volume integration | 3 | QM=0.1, Q1=1.5, Q2=0.6, QB/VDC=0.1, QW=0.1 |
| 900003 | Default hourglass type for shells in cortical bones | 8 | QM=0.1, Q1=1.5, Q2=0.6, QB/VDC=0.1, QW=0.1 |

**References:**


Dalstra, M., Huiskes, R., Odgaard, A., and van Erning, L. (1993). Mechanical and textural properties of pelvic trabecular bone. *Journal of Biomechanics* 26, 523–535. doi:10.1016/0021-9290(93)90014-6.

Ding, M. (2000). Age variations in the properties of human tibial trabecular bone and cartilage. *Acta Orthopaedica Scandinavica* 71, i–45. doi:10.1080/17453674.2000.11744841.

Engelbrektsson, K. (2011). Evaluation of material models in LS-DYNA for impact simulation of white adipose tissue.

Enns-Bray, W. S., Bahaloo, H., Fleps, I., Ariza, O., Gilchrist, S., Widmer, R., et al. (2018). Material mapping strategy to improve the predicted response of the proximal femur to a sideways fall impact. *Journal of the Mechanical Behavior of Biomedical Materials* 78, 196–205. doi:10.1016/j.jmbbm.2017.10.033.

Harris, R. I., Wallace, A. L., Harper, G. D., Goldberg, J. A., Sonnabend, D. H., and Walsh, W. R. (2000). Structural Properties of the Intact and the Reconstructed Coracoclavicular Ligament Complex. *Am J Sports Med* 28, 103–108. doi:10.1177/03635465000280010201.

Hewitt, J., Guilak, F., Glisson, R., and Vail, T. P. (2001). Regional material properties of the human hip joint capsule ligaments. *Journal of Orthopaedic Research* 19, 359–364. doi:10.1016/s0736-0266(00)00035-8.

Iatridis, J. C., Weidenbaum, M., Setton, L. A., and Mow, V. C. (1996). Is the nucleus pulposus a solid or a fluid? Mechanical behaviors of the nucleus pulposus of the human intervertebral disc. *Spine (Phila Pa 1976)* 21, 1174–1184. doi:10.1097/00007632-199605150-00009.

Iraeus, J., Brolin, K., and Pipkorn, B. (2020). Generic finite element models of human ribs, developed and validated for stiffness and strain prediction – To be used in rib fracture risk evaluation for the human population in vehicle crashes. *Journal of the Mechanical Behavior of Biomedical Materials* 106, 103742. doi:10.1016/j.jmbbm.2020.103742.

Ito, H., Song, Y., Lindsey, D. P., Safran, M. R., and Giori, N. J. (2009). The proximal hip joint capsule and the zona orbicularis contribute to hip joint stability in distraction. *Journal of Orthopaedic Research* 27, 989–995. doi:10.1002/jor.20852.

Kemper, A. R., McNally, C., and Duma, S. M. (2008). Dynamic tensile material properties of human pelvic cortical bone. *Biomedical sciences instrumentation* 44, 417–418.

Kopperdahl, D. L., and Keaveny, T. M. (1998). Yield strain behavior of trabecular bone. *Journal of Biomechanics* 31, 601–608. doi:10.1016/S0021-9290(98)00057-8.

Kunitomi, S., Yamamoto, Y., Kato, R., Antona-Makoshi, J., Konosu, A., Dokko, Y., et al. (2017). The Development of the Lower Extremity of a Human FE Model and the Influence of Anatomical Detailed Modelling in Vehicle-to-Pedestrian Impacts. in *International Research Council on the Biomechanics of Injury (IRCOBI)* Available at: http://www.ircobi.org/wordpress/downloads/irc17/pdf-files/62.pdf.

Li, Z., Alonso, J. E., Kim, J.-E., Davidson, J. S., Etheridge, B. S., and Eberhardt, A. W. (2006). Three-dimensional finite element models of the human pubic symphysis with viscohyperelastic soft tissues. *Annals of Biomedical Engineering* 34, 1452–1462. doi:10.1007/s10439-006-9145-1.

Loyd, A. M., Nightingale, R. W., Song, Y., Luck, J. F., Cutcliffe, H., Myers, B. S., et al. (2014). The response of the adult and ATD heads to impacts onto a rigid surface. *Accident Analysis & Prevention* 72, 219–229. doi:10.1016/j.aap.2014.06.022.

Manschot, J. F., and Brakkee, A. J. (1986). The measurement and modelling of the mechanical properties of human skin in vivo--II. The model. *J Biomech* 19, 517–521. doi:10.1016/0021-9290(86)90125-9.

Meyer, S., Bachelard, O., and De Grandi, P. (1998). Do bladder neck mobility and urethral sphincter function differ during pregnancy compared with during the non-pregnant state? *Int Urogynecol J* 9, 397–403. doi:10.1007/BF02199575.

Miller, J. A., Schultz, A. B., and Andersson, G. B. (1987). Load-displacement behavior of sacroiliac joints. *J Orthop Res* 5, 92–101. doi:10.1002/jor.1100050112.

Minns, R. J., and Hunter, J. A. A. (1976). The mechanical and structural characteristics of the tibio-fibular interosseous membrane. *Acta Orthopaedica Scandinavica* 47, 236–240. doi:10.3109/17453677608989725.

Mohammadkhah, M., Murphy, P., and Simms, C. K. (2016). The in vitro passive elastic response of chicken pectoralis muscle to applied tensile and compressive deformation. *Journal of the Mechanical Behavior of Biomedical Materials* 62, 468–480. doi:10.1016/j.jmbbm.2016.05.021.

Mukherjee, S., Chawla, A., Karthikeyan, B., and Soni, A. (2007). Finite element crash simulations of the human body: Passive and active muscle modelling. *Sadhana* 32, 409–426. doi:10.1007/s12046-007-0032-8.

Müller, S. S., Silvares, P. R. de A., Pereira, H. da R., Silva, M. A. de M., Sardenberg, T., and Leivas, T. P. (2004). Análise comparativa das propriedades mecânicas do ligamento da patela e do tendão calcâneo. *Acta Ortopédica Brasileira* 12, 134–140. doi:10.1590/s1413-78522004000300001.

Naseri, H. (2022). Calibration of Adipose tissue material properties in LS-DYNA. Chalmers University of Technology.

Östh, J., Bohman, K., and Jakobsson, L. (2020). Evaluation of Kinematics and Restraint Interaction when Repositioning a Driver from a Reclined to an Upright Position Prior to Frontal Impact using Active Human Body Model Simulations. in, 23.

Östh, J., Brolin, K., Svensson, M. Y., and Linder, A. (2016). A female ligamentous cervical spine finite element model validated for physiological loads. *Journal of Biomechanical Engineering* 138. doi:10.1115/1.4032966.

Östh, J., Mendoza-Vazquez, M., Linder, A., Svensson, M. Y., and Brolin, K. B. (2017a). The VIVA OpenHBM finite element 50th percentile female occupant model: Whole body model development and kinematic validation. in *IRCOBI conference proceedings*, 443–466.

Östh, J., Mendoza-Vazquez, M., Sato, F., Svensson, M. Y., Linder, A., and Brolin, K. (2017b). A female head–neck model for rear impact simulations. *Journal of Biomechanics* 51, 49–56. doi:10.1016/j.jbiomech.2016.11.066.

Panzer, M. B., and Cronin, D. S. (2009). C4–C5 segment finite element model development, validation, and load-sharing investigation. *Journal of Biomechanics* 42, 480–490. doi:10.1016/j.jbiomech.2008.11.036.

Peña, E., Calvo, B., Martínez, M. A., and Doblaré, M. (2006). A three-dimensional finite element analysis of the combined behavior of ligaments and menisci in the healthy human knee joint. *Journal of Biomechanics* 39, 1686–1701. doi:10.1016/j.jbiomech.2005.04.030.

Rater, J.-F. (2013). Thorax soft tissue response for validation of human body models and injury prediction.

Reilly, D. T., and Burstein, A. H. (1975). The elastic and ultimate properties of compact bone tissue. *Journal of Biomechanics* 8, 393–405. doi:10.1016/0021-9290(75)90075-5.

Roberts, C. R., Rains, J. K., Paré, P. D., Walker, D. C., Wiggs, B., and Bert, J. L. (1997). Ultrastructure and tensile properties of human tracheal cartilage. *Journal of Biomechanics* 31, 81–86. doi:10.1016/S0021-9290(97)00112-7.

Robinson, D. L., Kersh, M. E., Walsh, N. C., Ackland, D. C., de Steiger, R. N., and Pandy, M. G. (2016). Mechanical properties of normal and osteoarthritic human articular cartilage. *Journal of the mechanical behavior of biomedical materials* 61, 96–109. doi:10.1016/j.jmbbm.2016.01.015.

Schubert, A., Erlinger, N., Leo, C., Iraeus, J., John, J., and Klug, C. (2021). Development of a 50th Percentile Female Femur Model: International Research Council on the Biomechanics of Injury 2021. *2021 IRCOBI Conference Proceedings*, 308–332.

Yang, K. H., and Kish, V. L. (1988). Compressibility measurement of human intervertebral nucleus pulposus. *Journal of Biomechanics* 21, 865. doi:10.1016/0021-9290(88)90059-0.

Yoganandan, N., Pintar, F. A., Stemper, B. D., Baisden, J. L., Aktay, R., Shender, B. S., et al. (2006). Trabecular bone density of male human cervical and lumbar vertebrae. *Bone* 39, 336–344. doi:10.1016/j.bone.2006.01.160.

Zajac, F. E. (1989). Muscle and tendon: properties, models, scaling, and application to biomechanics and motor control. *Crit Rev Biomed Eng* 17, 359–411.
